# Cembranolides and Related Constituents from the Soft Coral *Sarcophyton cinereum*

**DOI:** 10.3390/molecules27061760

**Published:** 2022-03-08

**Authors:** Chih-Hua Chao, Yi-Ju Chen, Chiung-Yao Huang, Fang-Rong Chang, Chang-Feng Dai, Jyh-Horng Sheu

**Affiliations:** 1 School of Pharmacy, China Medical University, Taichung 406040, Taiwan; chchao@mail.cmu.edu.tw; 2 Chinese Medicine Research and Development Center, China Medical University Hospital, Taichung 404332, Taiwan; 3 Department of Marine Biotechnology and Resources, National Sun Yat-sen University, Kaohsiung 80424, Taiwan; yijuvivian@gmail.com (Y.-J.C.); betty8575@yahoo.com.tw (C.-Y.H.); 4 Graduate Institute of Natural Products, Kaohsiung Medical University, Kaohsiung 80708, Taiwan; aaronfrc@kmu.edu.tw; 5 Institute of Oceanography, National Taiwan University, Taipei 10617, Taiwan; corallab@ntu.edu.tw; 6 Department of Medical Research, China Medical University Hospital, China Medical University, Taichung 404332, Taiwan

**Keywords:** *Sarcophyton cinereum*, cinerenolides A–D, cytotoxity, α,β-unsaturated ε-lactone

## Abstract

In an attempt to explore the bioactive metabolites of the soft coral *Sarcophyton cinereum*, three new cembranolides, cinerenolides A–C (**1**–**3**), and 16 known compounds were isolated and identified from the EtOAc extract. The structures of the new cembranolides were elucidated on the basis of spectroscopic analysis, and the NOE analysis of cinerenolide A (**1**) was performed with the assistance of the calculated lowest-energy molecular model. The relative configuration of cinerenolide C (**3**) was determined by the quantum chemical NMR calculation, followed by applying DP4+ analysis. In addition, the cytotoxic assays disclosed that some compounds exhibited moderate to potent activities in the proliferation of P388, DLD-1, HuCCT-1, and CCD966SK cell lines.

## 1. Introduction

Soft corals of the genus *Sarcophyton* are a dominant species in many coral reef areas [1,2,3]. This species is well known to be a prolific producer of structurally unique diterpenes, especially cembranoids. Some of the cembranoid-type compounds have been found to be associated with coral reproduction [4,5]. Previous investigation of the *Sarcophyton* species has produced metabolites with diverse bioactivities, including anti-viral [6], anti-inflammatory [7,8,9,10,11], and cytotoxic activities [8,9,10,12]. As marine soft corals are a prolific source of bioactive cembranoids, investigations of promising structures with potent bioactivities have been persistently conducted in our laboratory. As part of our continuing search for bioactive structures from marine soft corals [7,8,9,10,11,12], the chemical constituents from the soft coral *Sarcophyton cinereum* are investigated in this study. Herein, we report the isolation and structural elucidation of three new cembranolides with an α,β-unsaturated ε-lactone (**1**–**3**), as well as 16 related cembranoids (**4**–**19**). Additionally, their cytotoxicities against a limited panel of cancer cell lines are reported.

## 2. Results

The EtOAc extract from *S. cinereum* was separated repeatedly by column chromatography and HPLC to afford three new diterpenoids (**1**–**3**) and 16 known compounds, which were identified as sarcophytonoxide E (**4**) [13], sarcomililatins A and B (**5** and **6**) [14], 2-[(*E*,*E*,*E*)-7′,8′-epoxy-4′,8′,12′-trimethylcyclotetradeca-1′,3′,11′-trienyl]propan-2-ol (**7**) [15], sarcophytonolide F (**8**) [16], cherbonolide L (**9**) [17], (+)-(2*S*)-isosarcophine (**10**) [18], (–)-(2*R*)-isosarcophine (**11**) [19], sarcophytonoxide A (**12**) [13], sartrolide C (**13**) [20], ketoemblide (**14**) [21], isosarcophytonolide D (**15**) [22], glaucumolides A and B (**16** and **17**) [23,24], and bistrochelides A and B (**18** and **19**) [24]. 

**Figure 1:**
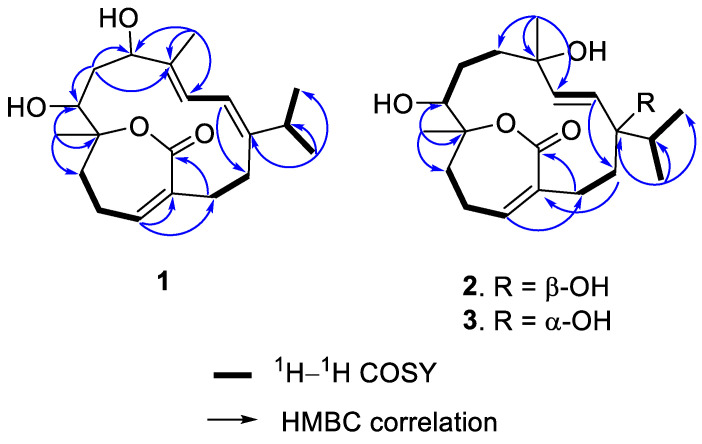
Selected ^1^H–^1^H COSY (▬) and HMBC (→) correlations of **1**–**3**.

**Figure 2:**
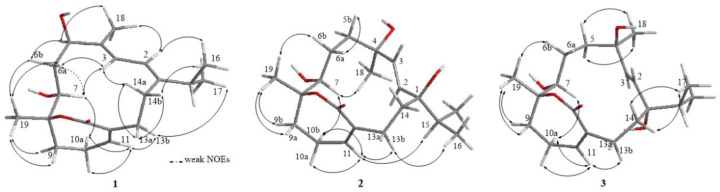
Selected NOE correlations of **1**–**3**.

The molecular formula of compound **1** was established as C_20_H_30_O_4_ by the analysis of its NMR data and HRESIMS. Its NMR data indicated that it is quite similar to the reduction products of sarcophytolide [21,25] (Table 1 and Table 2); however, a secondary hydroxyl group is observed at *δ*_C_ 73.0 (CH) and *δ*_H_ 4.19 (1H, ddd, *J* = 11.0, 8.0, 4.0 Hz, H-5) for **1**, revealing that one of the CH_2_ group in the cembranolide scaffold should be replaced by a hydroxy-containing methine. The HMBC correlations from H_3_-18 to C-3, C-4, and C-5 and from H_2_-6 to C-4, C-5, and C-7 indicated that the hydroxyl group was attached at C-5, as shown in Figure 1. Furthermore, two hydroxyl protons at *δ*_H_ 1.38 (1H, d, *J* = 8.0 Hz) and 1.24 (1H, d, *J* = 8.4 Hz) also supported the presence of two hydroxyl groups. The *E* geometry for the Δ^1^ and Δ^3^ double bonds was determined by the observation of NOE correlations (NOEs) of H-2 with both H_3_-18 and H_3_-16, and H-14a with H-3. The 7S*, 8R*-configuration was deduced from the NOEs of H_3_-19/H_2_-9, H_3_-19/H_2_-6, and H-7/H-10a (Figure 2). H-3 showed NOEs with both H-7 and H-6a, and H-5 had NOEs with both H_3_-18 and H-6, revealing a 5R*,7S*-configuration for C-5 and C-7 stereogenic centers (Supplementary Materials, Figures S1–S9).

**Scheme 1:**
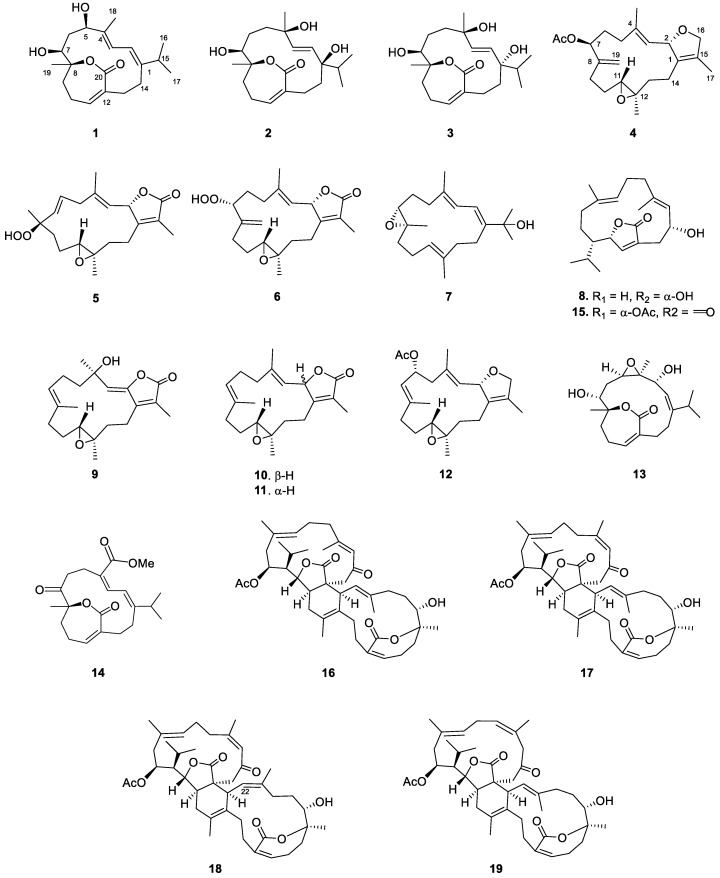
Structures of compounds **1**–**19**.

**Figure 3:**
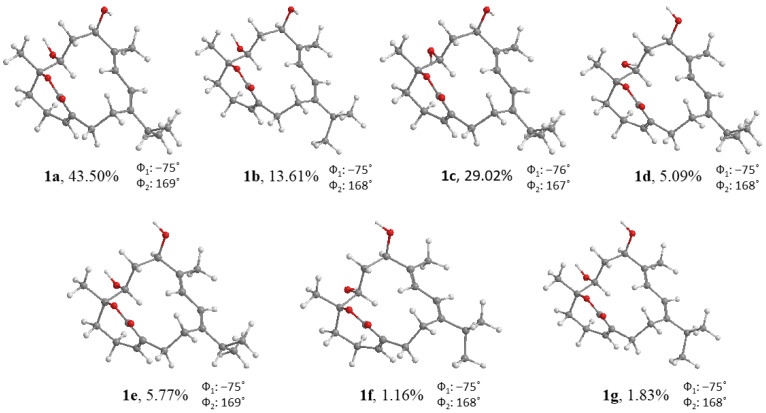
Low-energy conformers, populations, and dihedral angles (Φ_1_ (H7-C7-C6-H6*_pro-S_*) and Φ_2_ (H7-C7-C6-H6*_pro-R_*)) of 7S*, 8R*-**1** at CAM-B3LYP/6-31+G(d,p) IEFPCM (CHCl_3_) level of theory.

**Figure 4:**
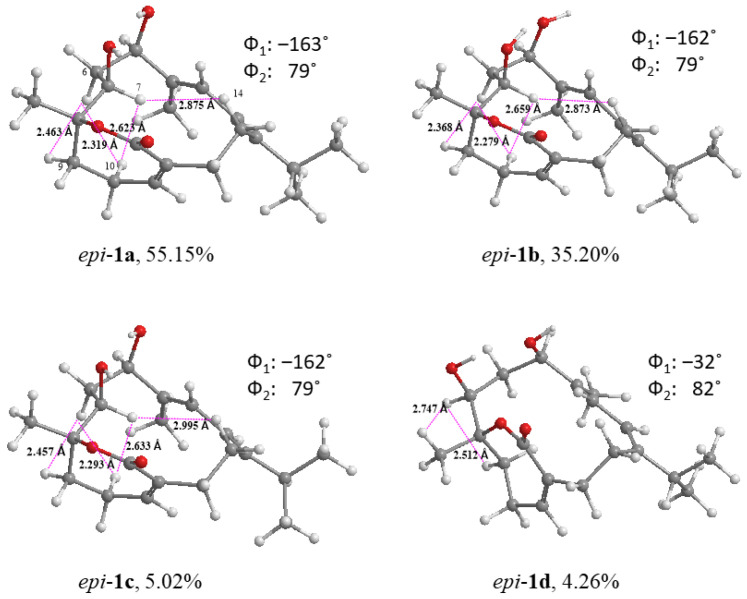
Low-energy conformers, populations, key proton-proton distances, and dihedral angles (Φ_1_ (H7-C7-C6-H6*_pro-S_*) and Φ_2_ (H7-C7-C6-H6*_pro-R_*)) of 7R*, 8R*-**1** (*epi*-**1**) at CAM-B3LYP/6-31+G(d,p) IEFPCM (CHCl_3_) level of theory.

As the known synthetic analogues possessing 7*S*, *8R* and 7*R*, *8R* configurations, which are derived from ketoemblide and sarcophytolide, have similar coupling patterns at H-7 (br d, *J* = 9.5–10.0 Hz for 7S*, 8R* and dd, *J* = 11.0, 2.5 Hz for 7R*, 8R*) [21], a detailed comparison between two computational models of **1** (7S*, 8R*-**1** and 7R*, 8R*-**1**) derived from DFT calculations was performed. A conformational search for both diastereomers of **1** was performed using the Merk Molecular Force Field (MMFF) calculation in Spartan’16 software. The resulting conformers within 5 kcal/mol were further subjected for geometry optimization and frequency calculation at the CAM-B3LYP/6-31+G(d,p) level with the integral equation formalism polarizable continuum model (IEFPCM)/CHCl_3_ in Gaussian 09 software [26], which generated seven conformers for 7S*, 8R*-**1** (Figure 3) and four for 7R*, 8R*-**1** (Figure 4) with Boltzmann populations over 1%. The conformers **1a**–**1g** of 7S*, 8R*-**1** (Figure 3) have almost the same conformation in the 14-membering carbon fragment, and differences were observed at the rotations of hydroxyl and isopropyl groups, which were quite similar to the model generated by the analysis of NOEs (Figure 2). On the other hand, four lower-energy conformers (*epi*-**1a**–**1d**) were obtained for another possible diastereomer, 7R*, 8R*-**1** (*epi*-**1**) (Figure 4). It is interesting that *epi*-**1a**–**1c**, accounting for 95.37% of the overall population, also possess an almost identical conformation for the 14-membering-ring skeleton. Although 7S*, 8R*-**1** and 7R*, 8R*-**1** have different arrangement neighboring the C-7 stereogenic center, the dihedral angles (Φ) of H-7 to H_2_-6 in the two possible diastereomers (7R*, 8R*-**1** and 7S*, 8R*-**1**) were quite similar, which could be the reason that the aforementioned 7*S*, *8R* and 7*R*, *8R* analogues derived from ketoemblide and sarcophytolide possess similar coupling patterns [21]. Similar to that of 7S*, 8R*-**1**, the distance of H-7/H-10 in *epi*-**1a**–**1c** (7R*, 8R*-**1**, Figure 4) is lower than 3Å, revealing that H-7 should have NOE enhancement with H-10 in both 7S*, 8R*-**1** and 7R*, 8R*-**1**; thus, this correlation could not be used as crucial NOEs to determine the C-7 configuration. In addition, the distances of H-6/H-9, H-6/H-10, and H-7/H-14 in 7R*, 8R*-**1** are also lower than 3 Å (Figure 4), implying that these protons are expected to have NOEs; however, these correlations were not found in compound **1**, which further supports the 7S*, 8R* configuration for **1**. A comparison of the proton chemical shift of H_3_-19 (*δ*_H_ 1.41 s) in **1** to the literature data (1.38–1.41 ppm for 7*S*, 8*R* analogues; 1.13–1.16 ppm for 7*R*, 8*R* analogues) [21] also confirmed the relative configurations of C-7 and C-8 to be 7S* and 8R*, respectively. Accordingly, the structure of **1** was determined as shown (Scheme 1).

Compound **2** was obtained as a white powder and suggested a molecular formula of C_20_H_32_O_5_ based on the molecular ion peak [M + H]^+^ at *m*/*z* 375.2141 in the (+)-HR-ESI-MS (calculated for C_20_H_32_O_5_Na, 375.2142). Inspection of the overall ^1^H and ^13^C NMR data revealed signals characteristic of an α,β-conjugated carboxylate system (*δ*_C_ 144.8 (CH, C-11), 134.8 (C, C-12), and 170.4 (C, C-20); *δ*_H_ 6.55 t (*J* = 3.9 Hz, H-11)), and a disubstituted double bond (*δ*_C_ 135.1 (CH × 2, C-2); *δ*_H_ 5.40 and 5.53 (both 1H, d, *J* = 16.4 Hz, H-2 and H-3)). The former was evidenced by the IR absorption band at 1653 cm^–1^. Additionally, two hydroxy-containing quaternary carbons (*δ*_C_ 77.0 (C, C-1); 74.9 (C, C-4)), one hydroxy-containing methine (*δ*_C_ 71.7 (CH, C-7); *δ*_H_ 3.92 (1H, d, J = 10.8 Hz, H-7)), and a down-field shifted quaternary carbon (*δ*_C_ 86.1 (C, C-8)) were evidenced. Considering the molecular formula and the above functionality, the structure of **2** should be bicyclic.

In an extensive analysis of ^1^H-^1^H COSY, HSQC, and HMBC spectra (Figure 1), the planar structure of **2** was established and found to be quite similar to sartrolide D (Supplementary Materials, Table S1) [20]. A large coupling constant of 16.4 Hz indicated the *E* geometry for the Δ^1^ double bond. The same 7S*, 8R*-configuration as **1** was assigned for **2,** as they showed similar NOEs neighboring the C-7 and C-8 stereogenic centers (Figure 2). Furthermore, the NOEs of H-7/H_3_-18 and H-11/H-15 indicated that H_3_-18 and the isopropyl group were cofacial (Supplementary Materials, Figures S10–S17). Accordingly, the structure of **2** was determined as shown (Scheme 1). 

Compound **3** was also obtained as a white powder with the same molecular formula, determined to be C_20_H_32_O_5_ from HRESIMS, as that of **2**. Their NMR data were quite similar; however, differences were observed for the chemical shifts around C-1 and C-4. Its planar structure was confirmed by an analysis of the 1D and 2D NMR data (Figure 1). Compound **3** has the same 7S*, 8R* configuration based on similar NOEs neighboring C-7 and C-8; however, the relative configurations of C-1 and C-4 remained unclear in an analysis of the NOEs (Figure 2) (Supplementary Materials, Figures S18–S25). Thus, the computational NMR data with DP4+ analysis [27,28] was applied for the establishment of the relative configuration of **3**. The four possible isomers with two hydroxyl groups at C-1 and C-4, respectively, 1α4β, 1β4α, 1α4α, and 1β4β, were subjected for chemical shift calculations at the MPW1PW91/6-31+G(d,p)//B3LYP/6-31G(d) level with the polarizable continuum model (PCM). Then, the calculated NMR chemical shifts for the four possible isomers were compared with the experimental data of **3** and statistically analyzed using the DP4+ method, as shown in the Supplementary Materials. As a result, the conformer 1α4β was found to have a probability of 100% (Table 3) (Supplementary Materials, Tables S2–S6), suggesting a 1S*, 4R* configuration for **3**.

As marine cembranoids have been proven to show a broad spectrum of biological activities, including anti-inflammatory [29], anti-oxidant [30], and cytotoxicity activities [30,31], compounds **2**–**19** were evaluated for their proliferation activities toward the P388, DLD-1, HuCCT-1, and CCD966SK cell lines (Table 4). Among the tested compounds, **18** exhibited the most potent activity to inhibit the proliferation of the HuCCT-1 cell with an IC_50_ value of 2.0 μM, which is comparable to the positive control, doxorubicin (HuCCT-1, IC_50_ = 1.9 μM), whereas compound **18** showed moderate anti-proliferation activity to P388 and DLD-1, with IC_50_s of 10.6 and 9.9 μM, respectively. In addition, compounds **5** and **6** were also found to show moderate activities toward P388 cells with IC_50_s of 15.2 and 11.8 μM, respectively. The other compounds, as shown in Table 4, were found to possess weak activities toward the above four cancer cell lines. In a comparison of the biological data between biscembranolids (**16**–**19**), we found that the Δ^22^ double bond with a *Z* geometry in compound **18** dramatically and selectively increased the anti-proliferation activity toward HuCCT-1 cell line.

## 3. Materials and Methods

### 3.1. General Experimental Procedures

Optical rotations were determined with a JASCO P1020 digital polarimeter. IR spectra were taken on a JASCO FT/IR-4100 spectrometer. The NMR spectra were recorded on a Varian 400MR FT-NMR instrument at 400 MHz for ^1^H and 100 MHz for ^13^C, and on a Varian Unity INOVA 500 FT-NMR spectrometer at 500 MHz for ^1^H and 125 MHz for ^13^C in CDCl_3_. LR- and HR-ESIMS were measured with a Bruker APEX II mass spectrometer. Silica gel 60 (230–400 mesh, Merck, Darmstadt, Germany) and SiliaBond C18 silica gel (40–63 µm, 60 Å, 17% carbon loading, SiliCycle, Québec, QC, Canada) were used for column chromatography. Precoated silica gel plates (Kieselgel 60 F254, Merck, Darmstadt, Germany) and precoated silica gel RP-18 plates (Kieselgel 60 F254S, Merck, Darmstadt, Germany) were used for TLC analysis. 

### 3.2. Animal Material

The animal material, *S. cinereum*, was collected from the coral reef of Xiaoliuqiu island of Taiwan in 2012. The specimen was identified by Prof. C.-F. Dai. A voucher specimen (specimen no. sheuCYJ-001) was deposited in the Department of Marine Biotechnology and Resources, National Sun Yat-sen University, Kaohsiung 804, Taiwan.

### 3.3. Extraction and Isolation

The animal tissues (107.4 g) were freeze-dried, minced, and extracted exhaustively with 700 mL of EtOAc for 2 h at room temperature, and the extraction was repeated 12 times. The concentrated EtOAc layer (14.6 g) was fractionated using silica gel column chromatography (CC) with a gradient system, comprising mixtures of hexane–EtOAc (100:1 to 1:100) and EtOAc–MeOH (100:1 to 80:20), to yield 22 fractions. Fraction 8 was fractionated by silica gel CC (eluent, hexane–acetone, 4:1) and semipreparative RP-18 HPLC (eluent, MeOH–H_2_O, 4:1) to give **4** (1.5 mg) and **7** (5.1 mg). Compounds **5** (2.3 mg), **6** (1.4 mg), **12** (0.8 mg), and **15** (2.5 mg) were yielded from fraction 10 by silica gel CC (eluent, hexane–acetone, 5:1), and by semipreparative RP-18 HPLC (eluent, ACN–H_2_O, 1:1). Fraction 11 was purified by semipreparative RP-18 HPLC (eluent, ACN–H_2_O, 1.25:1) to yield compounds **9** (1.0 mg), **10** (67.1 mg), and **11** (0.7 mg). Fraction 13 was subjected to silica gel CC (hexane–acetone, 4.5:1), followed by RP-18 HPLC (eluent, MeOH–H_2_O, 2.5:1), to obtain **8** (1.0 mg) and **14** (2.0 mg). Fraction 15 was separated with silica gel CC (eluent, hexane–acetone, 5:1) to yield two subfractions (F15-1 and F15-2). The F15-1 fraction was subjected for semipreparative RP-18 HPLC (eluent, MeOH–H_2_O, 5.5:1) to obtain **16** (2.0 mg), **17** (57.0 mg), **18** (1.0 mg), and **19** (15.0 mg). Two subfractions (F17-1 and F17-2) were obtained using silica gel CC (hexane–acetone, 2.5:1), and fraction 17-1 was separated by semipreparative RP-18 HPLC (eluent, MeOH–H_2_O, 1:1) to yield **2** (3.4 mg) and **3** (2.1 mg). In addition, compound **1** (1.0 mg) was purified by semipreparative RP-18 HPLC (eluent, MeOH–H_2_O, 2:1) from subfraction F17-2.

Cinerenolide A (**1**): white powder; [*α*]^25^_D_ +4.4 (*c* 0.97, CHCl_3_); IR (KBr) *v*_max_ 3416, 2960, 2926, 1653, 1452, 1236, 1070 cm^−1^; ^13^C and ^1^H NMR data, see Table 1; ESIMS *m/z* 375 [M + Na]^+^; HRESIMS *m/z* 375.2141 [M + Na]^+^ (calcd for C_20_H_32_O_5_Na, 375.2142). 

Cinerenolide B (**2**): white powder; [*α*]^25^_D_ +24.3 (*c* 0.60, CHCl_3_); IR (KBr) *v*_max_ 3434, 2917, 2859, 1660, 1376, 1018 cm^−1^; ^13^C and ^1^H NMR data, see Table 1; ESIMS *m/z* 375 [M + Na]^+^; HRESIMS *m/z* 375.2139 [M + Na]^+^ (calcd for C_20_H_32_O_5_Na, 375.2142).

Cinerenolide C (**3**): colorless oil; [*α*]^25^_D_ +71.4 (*c* 0.37, CHCl_3_); IR (KBr) *v*_max_ 3416, 2960, 2926, 1653, 1452, 1235, 1070 cm^−1^; ^13^C and ^1^H NMR data, see Table 1; ESIMS *m/z* 357 [M + Na]^+^; HRESIMS *m/z* 357.2034 [M + Na]^+^ (calcd for C_20_H_30_O_4_Na, 357.2036).

### 3.4. Computational Method

The conformers found at the MMFF force field using Spartan’16 were selected within a 5 kcal/mol energy window. Twelve conformers were selected for **1** and subjected for geometry optimizations and frequency calculations at the CAM-B3LYP/6-31+G(d,p) level of theory with IEFPCM in CHCl_3_. The populations were calculated based on the Gibbs free energy obtained in the aforementioned frequency calculation. For DP4+ analysis, systematic conformational searches were performed for the possible isomers 1α4β, 1β4α, 1α4α, and 1β4β of **3**, using the MMFF force field in gas phase. All conformers within a 5 kcal/mol energy window were subjected for geometry optimizations and frequency calculations at the B3LYP/6-31+G(d) level in gas phase. The conformers within 2 kcal/mol from the global minimum were subjected to chemical shift calculations using the gauge-independent atomic orbital (GIAO) method at the mPW1PW91/6-31G+(d,p)//B3LYP/6-31G(d) level with PCM/MeOH. The Boltzmann-weighted NMR data of the four isomers and the experimental data of **3** were used for DP4+ probability analysis using the Excel sheet provided by Grimblat et al. [27,28].

### 3.5. Cytotoxicity Assay

The assay was implemented according to the published protocols [32,33]. In brief, the Alamar Blue assay was performed for compounds **2**–**19** by treating them with P388, DLD-1, HuCC-T1, and CCD966SK cancer cells, which were commercially available from the American Type Culture Collection (ATCC). The test was performed in triplicate, and doxorubicin was used as a positive control.

## 4. Conclusions

In total, 3 new and 16 known compounds were isolated from the soft coral *S. cinereum*. In the cytotoxicity assay, compound **18** was found to show potent and selective activity toward HuCCT-1 cell line, which is close to the control group, doxorubicin. The relative configuration of **1** was determined by an analysis of NOEs and by comparing the computational conformers with those of its possible epimer. The assignment of the relative configurations of **3**, with the lack of crucial NOEs, was successfully attained by the assistance of quantum chemical NMR calculation and the DP4+ method. In this work, it was also found that some cembranolides were not so flexible, and they could be readily assigned the relative configurations by a careful analysis of NOEs based on a computational model. In contrast to the flexible molecules, the assignment of relative configuration was hindered by a lack of useful NOE data neighboring the stereogenic center. For this case, the computational NMR data coupled with the DP4+ approach could provide an alternative to elucidate the relative configurations of stereogenic centers.

## Figures and Tables

**Table 1:** 1H NMR spectroscopic data of compounds **1**–**3**.

	1 ^a^	2 ^b^	2 ^c^	3 ^b^
No.	*δ*_H_ (*J* in Hz)	*δ*_H_ (*J* in Hz)	*δ*_H_ (*J* in Hz)	*δ*_H_ (*J* in Hz)
2	6.13 d (11.6)	5.40 d (16.4)	5.37 d (16.4)	5.44 d (16.0)
3	5.82 d (11.6)	5.53 d (16.4)	5.52 d (16.4)	5.43 d (16.0)
5	4.19 (11.0, 8.0, 4.0)	1.84 m	1.98 m	1.74 m
		1.73 m	1.82 m	
6	2.30 m	1.66 m	1.66 m	1.82 m
	1.89 m	1.46 m	1.66 m	1.37 m
7	4.11 dd (9.2, 8.4)	3.92 d (10.8)	3.75 d (10.0)	3.79 d
9	2.21 m	2.24 m	2.23 m	2.20 m
	2.06 m	1.95 m	2.00 m	1.89 m
10	2.58 m	2.69 m	2.53 m	2.67 m
	2.40 m	2.52 m	2.50 m	2.45 m
11	6.08 t (3.6)	6.55 t (3.9)	6.51 t (5.0)	6.49 s
13	3.17 t (12.2)	2.90 td (12.0, 4.0)	3.09 m	2.72 m
	1.84 m	2.22 m	2.50 m	2.38 m
14	2.57 td (12.2, 8.0)	1.92 m	1.90 m	2.27 m
	2.13 m	1.79 m	1.81 m	1.67 m
15	2.33 m	1.70 m	1.80 m	1.60 m
16	1.10 d (6.8)	0.89 d (6.8)	0.85 d (6.8)	0.87 d (6.4)
17	1.06 d (6.8)	0.81 d (6.8)	0.80 d (6.8)	0.83 d (6.4)
18	1.92 s	1.26 s	1.30 s	1.25 s
19	1.41 s	1.29 s	1.31 s	1.26 s
5-OH	1.38 d (8.0)			
7-OH	1.24 d (8.4)			

^a^ Spectra recorded at 400 MHz in CDCl_3_; ^b^ spectra recorded at 400 MHz in CD_3_OD; ^c^ spectra recorded at 500 MHz in CDCl_3._

**Table 2:** ^13^C NMR spectroscopic data of compounds **1**–**3**.

	1 ^a^	2 ^b^	2 ^c^	3 ^b^
No.	*δ*_C_ (mult.)	*δ*_C_ (mult.)	*δ*_C_ (mult.)	*δ*_C_ (mult.)
1	149.9 (C)	77.0 (C)	74.5 (C)	77.1 (C)
2	118.5 (CH)	135.1(CH)	134.8 (CH)	132.4 (CH)
3	123.0 (CH)	135.1(CH)	133.4 (CH)	135.1(CH)
4	133.3 (C)	74.9 (C)	73.8 (C)	74.0 (C)
5	73.0 (CH)	37.5 (CH_2_)	34.0 (CH_2_)	36.9 (CH_2_)
6	36.0 (CH_2_)	25.6 (CH_2_)	25.6 (CH_2_)	25.2 (CH_2_)
7	67.5 (CH)	71.7 (CH)	72.2 (CH)	70.2 (CH)
8	83.0 (C)	86.1 (C)	83.9 (C)	85.7 (C)
9	34.1 (CH_2_)	35.3 (CH_2_)	36.6 (CH_2_)	35.8 (CH_2_)
10	27.3 (CH_2_)	28.4 (CH_2_)	27.9 (CH_2_)	28.3 (CH_2_)
11	140.5 (CH)	144.8 (CH)	144.0 (CH)	145.2 (CH)
12	133.2 (C)	134.8 (C)	134.2 (C)	132.6 (C)
13	37.4 (CH_2_)	32.9 (CH_2_)	32.0 (CH_2_)	35.6 (CH_2_)
14	27.2 (CH_2_)	39.0 (CH_2_)	38.0 (CH_2_)	36.8 (CH_2_)
15	35.7 (CH)	38.4 (CH)	40.1 (CH)	43.4 (CH)
16	22.1 (CH_3_)	16.8 (CH_3_)	16.7 (CH_3_)	17.1 (CH_3_)
17	22.8 (CH_3_)	17.4 (CH_3_)	16.9 (CH_3_)	18.0 (CH_3_)
18	17.2 (CH_3_)	31.9 (CH_3_)	32.0 (CH_3_)	32.5 (CH_3_)
19	22.0 (CH_3_)	22.5 (CH_3_)	21.7 (CH_3_)	22.7 (CH_3_)
20	166.5 (C)	170.4 (C)	168.7 (C)	169.3 (C)

^a^ Spectra recorded at 100 MHz in CDCl_3_; ^b^ spectra recorded at 100 MHz in CD_3_OD; ^c^ spectra recorded at 125 MHz in CDCl_3_.

**Table 3:** DP4+ probabilities for possible isomers of compound **3**.

	**DP4+ (%)**			
	1α4β-**3**	1β4α-**3**	1α4α-**3**	1β4β-**3**
H	100.00%	0%	0%	0%
C	100.00%	0%	0%	0%
All data	100.00%	0%	0%	0%

**Table 4:** Anti-proliferation activities (IC_50_, μM) of **2**–**19**.

Compound	P388 ^a^	DLD-1 ^b^	HuCCT-1 ^c^	CCD966SK ^d^
**2**	>30	>30	>30	>30
**3**	>30	>30	>30	>30
**4**	>30	>30	>30	>30
**5**	15.2 ± 3.2	>30	>30	>30
**6**	11.8 ± 4.6	>30	>30	>30
**7**	>30	>30	>30	>30
**8**	>30	>30	>30	>30
**9**	>30	>30	>30	>30
**10**	>30	>30	>30	>30
**11**	>30	>30	>30	>30
**12**	>30	>30	>30	>30
**13**	>30	>30	>30	>30
**14**	>30	>30	>30	>30
**15**	>30	>30	>30	27.6 ± 7.8
**16**	16.7 ± 5.8	>30	19.1 ± 6.4	21.3 ± 5.8
**17**	22.8 ± 9.7	>30	>30	26.1 ± 10.3
**18**	10.6 ± 1.9	9.9 ± 1.0	2.0 ± 0.1	18.8 ± 6.9
**19**	>30	>30	>30	18.8 ± 6.8
Doxorubicin	0.69 ± 0.01	4.1 ± 0.7	1.9 ± 0.1	2.9 ± 0.4

^a^ mouse lymphoma. ^b^ human colorectal adenocarcinoma. ^c^ human intrahepatic cholangiocarcinoma. ^d^ human skin fibroblast.

## Data Availability

The data used to support the findings of this study are available from the corresponding author upon request.

## Supplementary Materials

The following are available online at https://www.mdpi.com/article/10.3390/molecules27061760/s1, Figures S1–S25: NMR (1D and 2D) and MS spectra of compounds **1**–**3**, Table S1: Comparison of NMR data between 2 and sartrolide D, Table S2: DP4+ analysis table for compound **3**, Tables S3–S6: Conformers and Boltzmann populations of isomers of **3**.
