# Supplementary material for: Cembranolides and Related Constituents from the Soft Coral Sarcophyton cinereum
Source: Molecules. 2022 Mar 8;27(6):1760. doi: 10.3390/molecules27061760 (PMC8956058; doi:10.3390/molecules27061760)
Supplement: Supplementary file 1 [file molecules-27-01760-s001.zip › molecules-1614405-supplementary.pdf]

Cembranolides and Related Constituents from the Soft Coral *Sarcophyton cinereum*

Chih-Hua Chao[1,2,†], Yi-Ju Chen[3,†], Chiung-Yao Huang[3], Fang-Rong Chang[4], Chang-Feng Dai[5] and Jyh-Horng Sheu[3,4,6,*]

1. School of Pharmacy, China Medical University, Taichung 406040, Taiwan; chchao@mail.cmu.edu.tw (C.-H. C.)

2. Chinese Medicine Research and Development Center, China Medical University Hospital, Taichung 404332, Taiwan

3. Department of Marine Biotechnology and Resources, National Sun Yat-sen University, Kaohsiung 80424, Taiwan; yijuvivian@gmail.com (Y.-J. C.); huangcy@mail.nsysu.edu.tw (C.-Y.H.)

4. Graduate Institute of Natural Products, Kaohsiung Medical University, Kaohsiung 80708, Taiwan; aaronfrc@kmu.edu.tw (F.-R. C.)

5. Institute of Oceanography, National Taiwan University, Taipei 10617, Taiwan; corallab@ntu.edu.tw (C.-F.D.)

6. Department of Medical Research, China Medical University Hospital, China Medical University, Taichung 404332, Taiwan

*Correspondence: sheu@mail.nsysu.edu.tw; Tel: +886-7-5252000 (ext. 5030); Fax: +886-7-5255020

† These authors contributed equally to this work

Figure S1. LR- and HR-ESIMS spectra of **1**.
Figure S2. ^{1}H NMR spectrum of **1**.
Figure S3. Partial ^{1}H NMR spectrum of **1**.
Figure S4. ^{13}C NMR spectrum of **1**.
Figure S5. DEPT spectrum of **1**.
Figure S6. HSQC spectrum of **1**.
Figure S7. HMBC spectrum of **1**.
Figure S8. COSY spectrum of **1**.
Figure S9. NOESY spectrum of **1**.
Figure S10. LR- and HR-ESIMS spectra of **2**.
Figure S11. ^{1}H NMR spectrum of **2**.
Figure S12. ^{13}C NMR spectrum of **2**.
Figure S13. DEPT spectrum of **2**.
Figure S14. HSQC spectrum of **2**.
Figure S15. HMBC spectrum of **2**.
Figure S16. COSY spectrum of **2**.
Figure S17. NOESY spectrum of **2**.
Figure S18. LR- and HR-ESIMS spectra of **3**.
Figure S19. ^{1}H NMR spectrum of **3**.
Figure S20. ^{13}C NMR spectrum of **3**.
Figure S21. DEPT spectrum of **3**.
Figure S22. HSQC spectrum of **3**.
Figure S23. HMBC spectrum of **3**.
Figure S24. COSY spectrum of **3**.
Figure S25. NOESY spectrum of **3**.
Table S1. Comparison of NMR data between **2** and sartrolide D.
Table S2. DP4+ analysis table for compound **3** (isomer 1: 1α4β-**3**; isomer 2: 1β4α-**3**; isomer 3: 1α4α-**3**; isomer 4: 1β4β-**3**).
Table S3. Conformers and Boltzmann populations of 1α4β-**3**.
Table S4. Conformer and Boltzmann population of 1β4α-**3**.
Table S5. Conformers and Boltzmann populations of 1α4α-**3**.
Table S6. Conformers and Boltzmann populations of 1β4β-**3**.

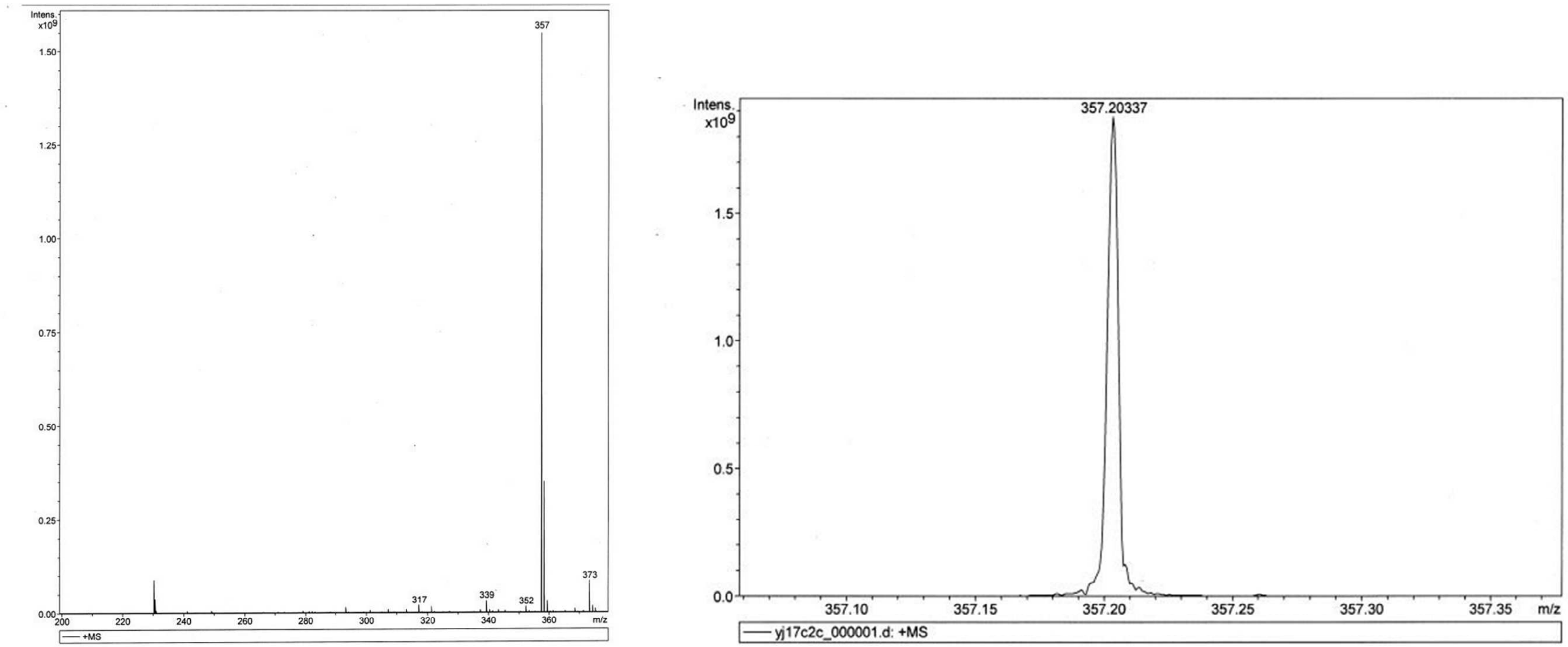

Figure S1. LR- and HR-ESIMS spectra of **1**.

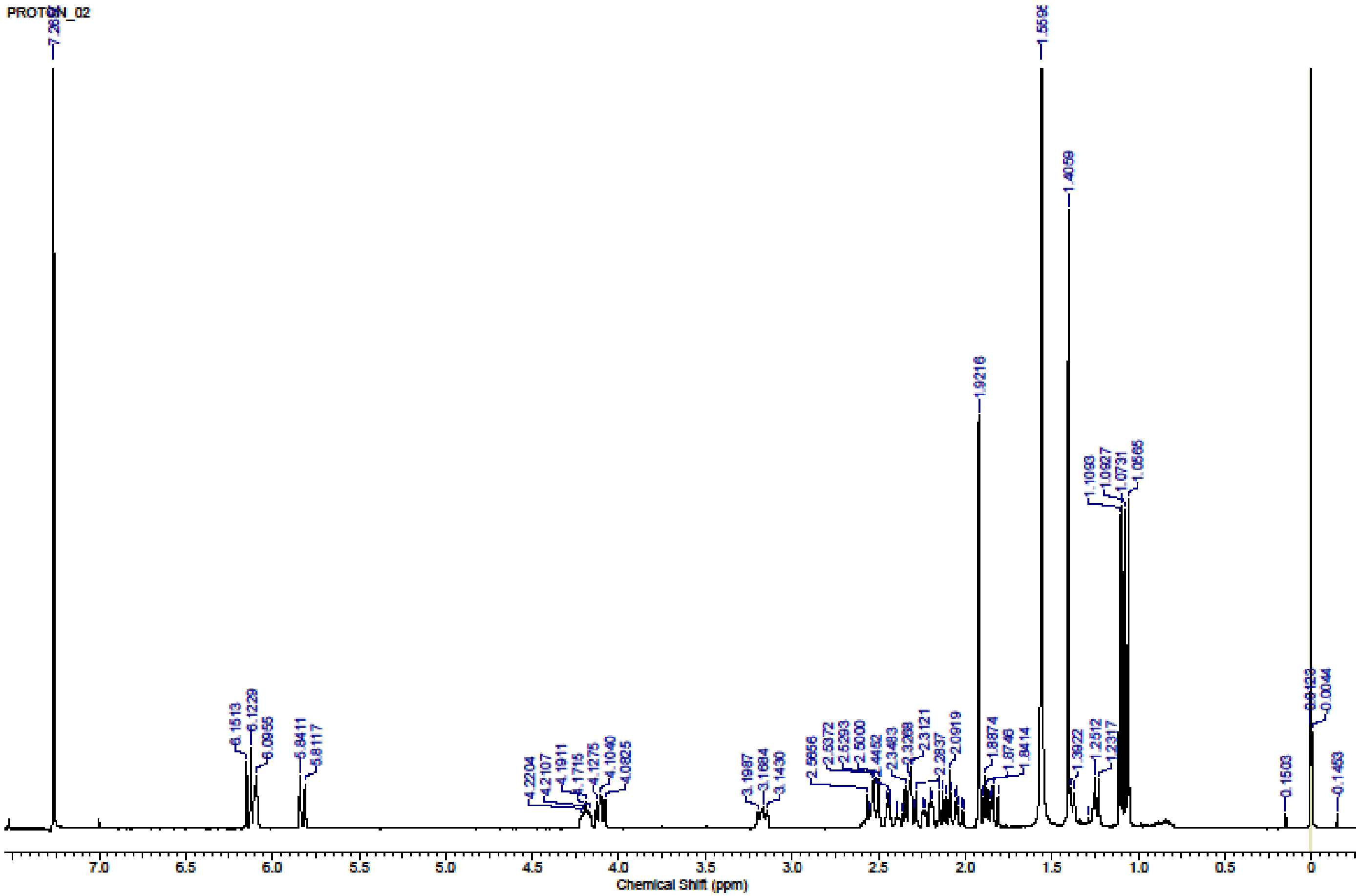

Figure S2. ^{1}H NMR spectrum of **1**.

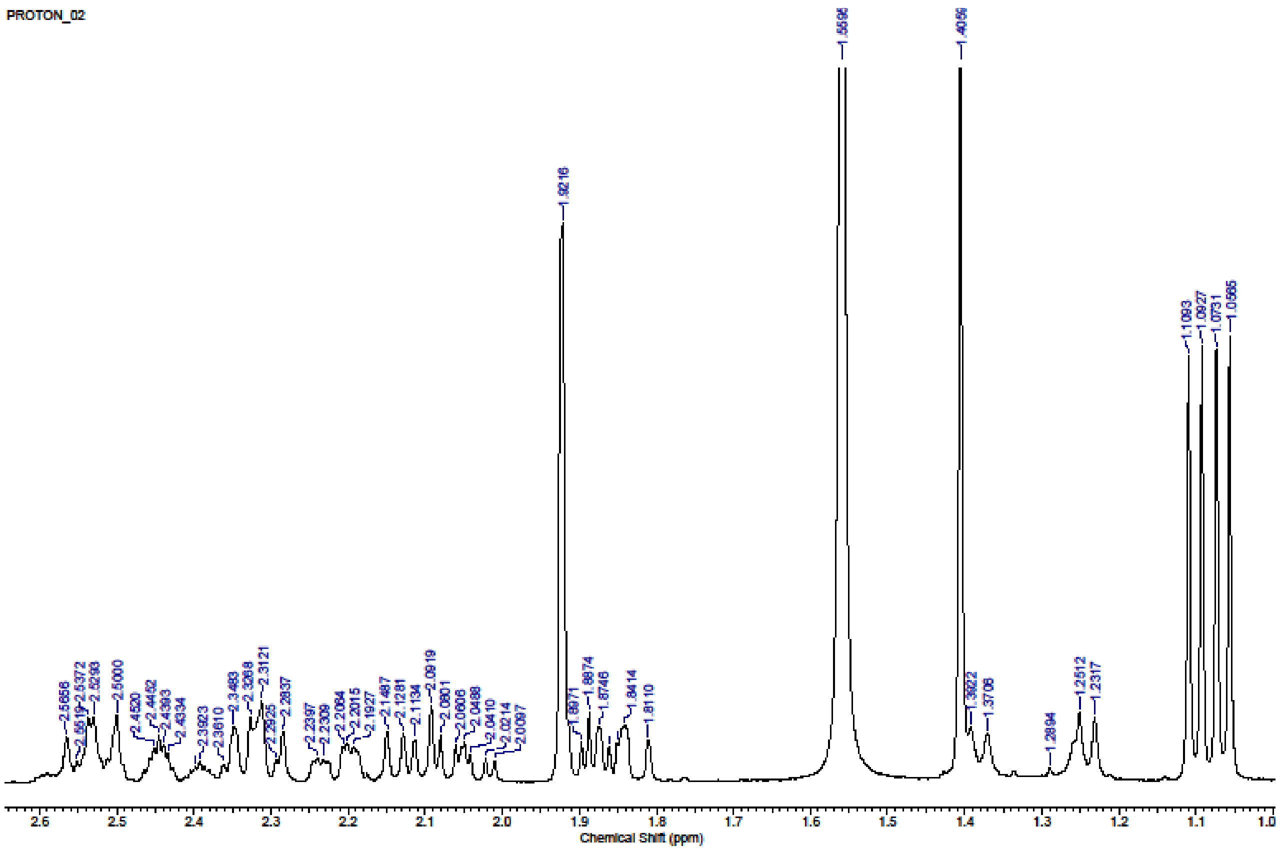

Figure S3. Partial ^{1}H NMR spectrum of **1**.

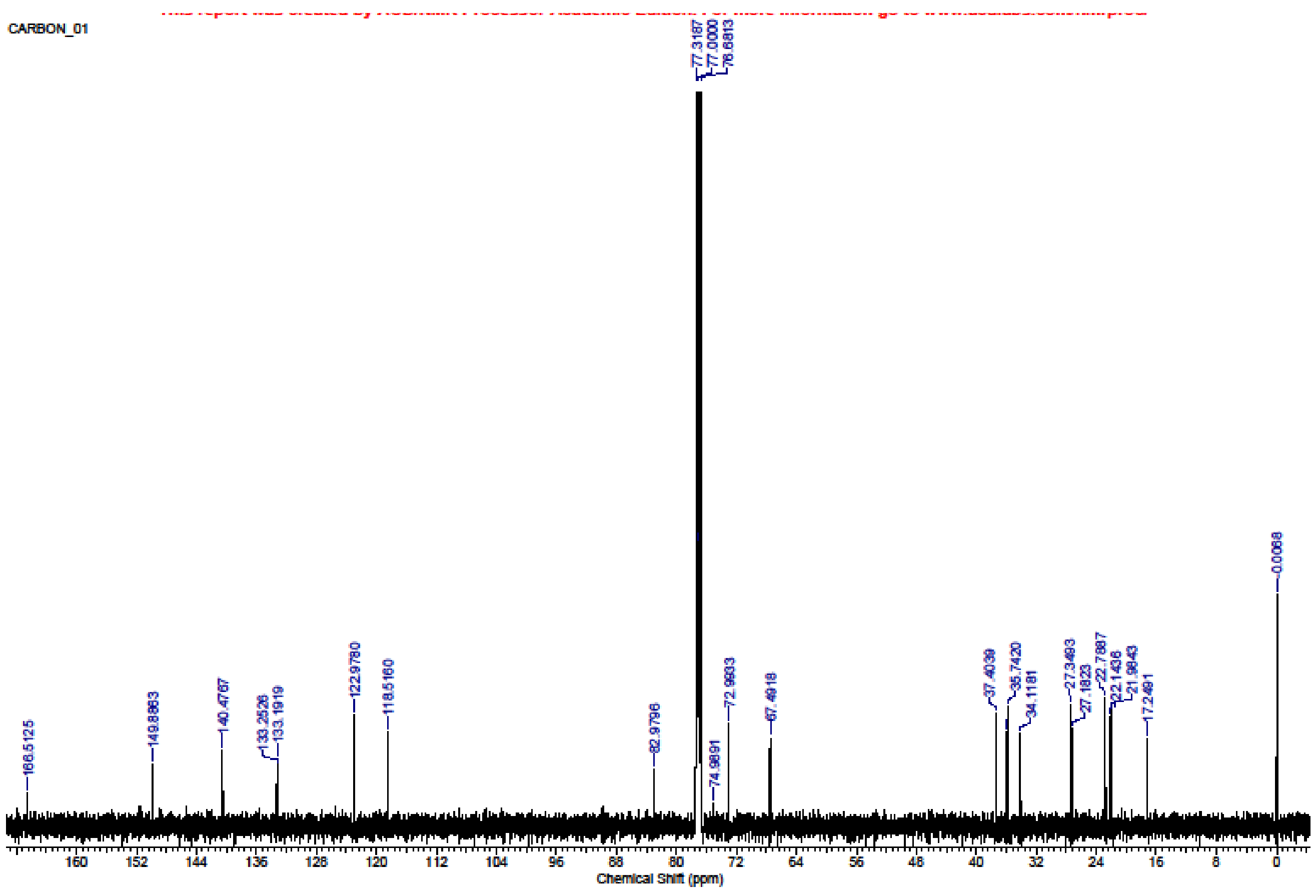

Figure S4. ^{13}C NMR spectrum of **1**.

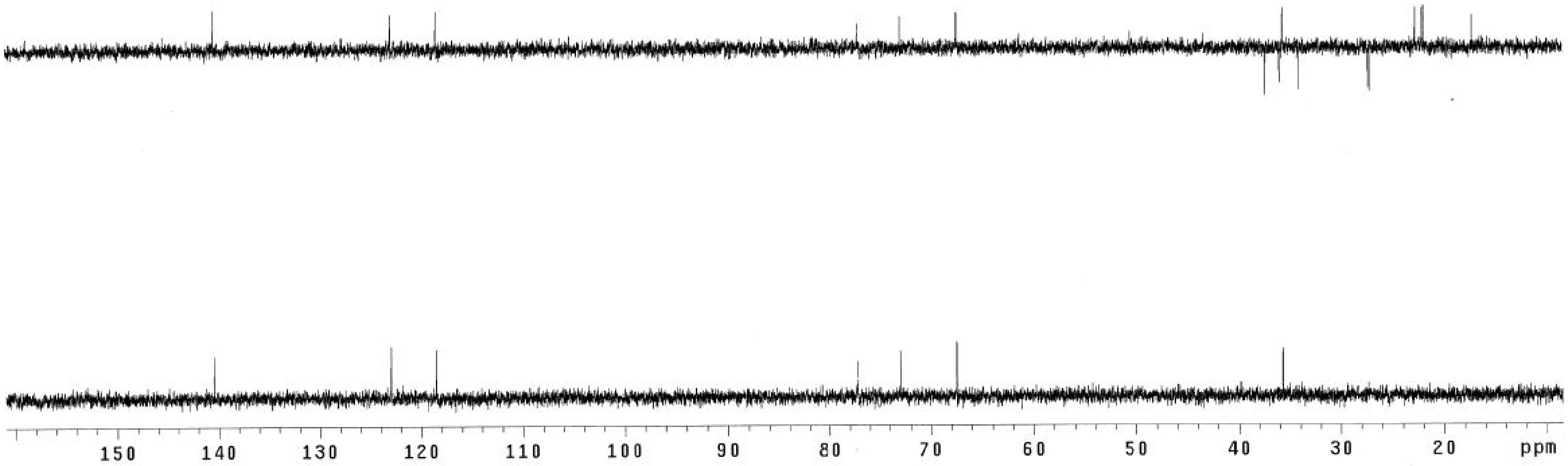

Figure S5. DEPT spectrum of **1**.

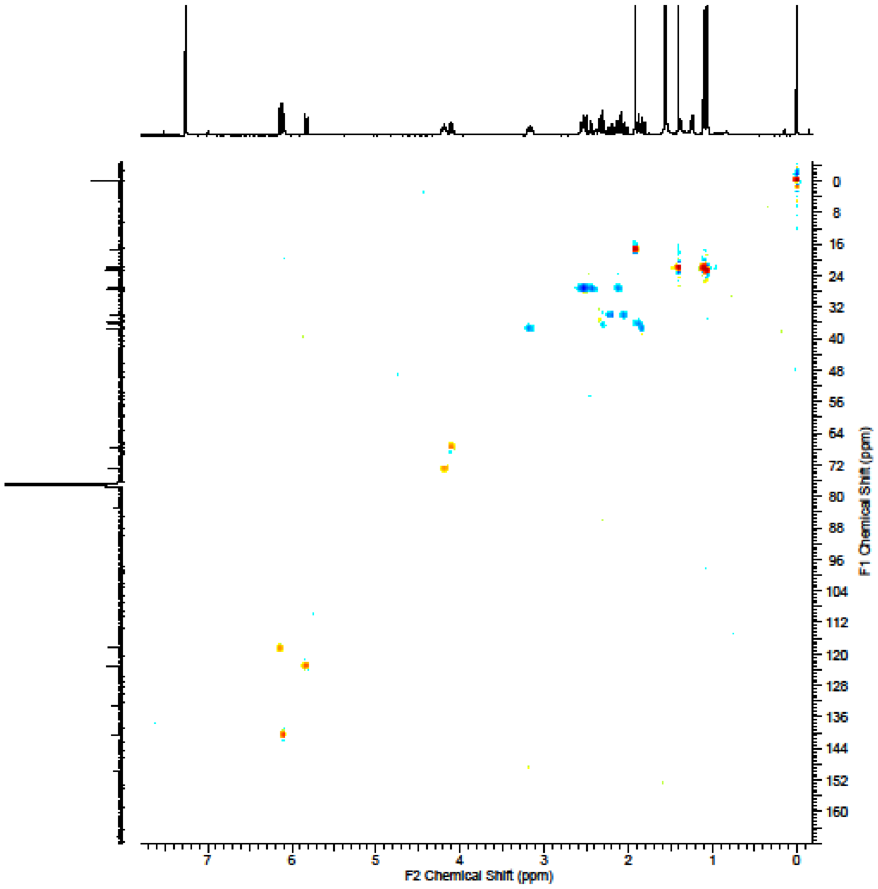

Figure S6. HSQC spectrum of **1**.

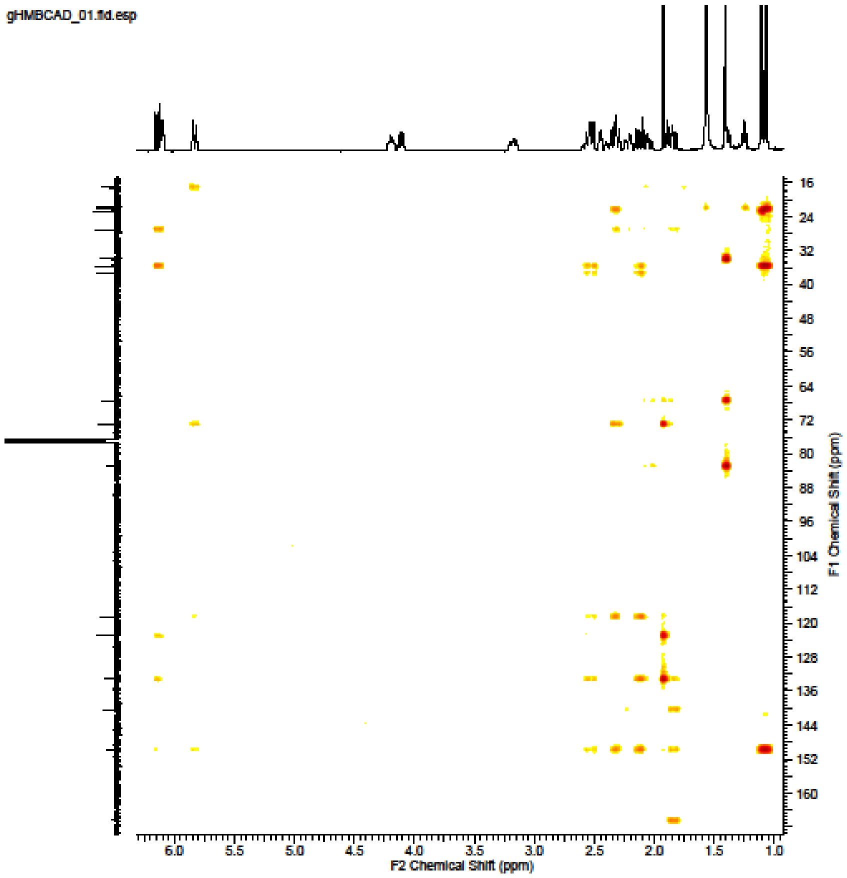

Figure S7. HMBC spectrum of **1**.

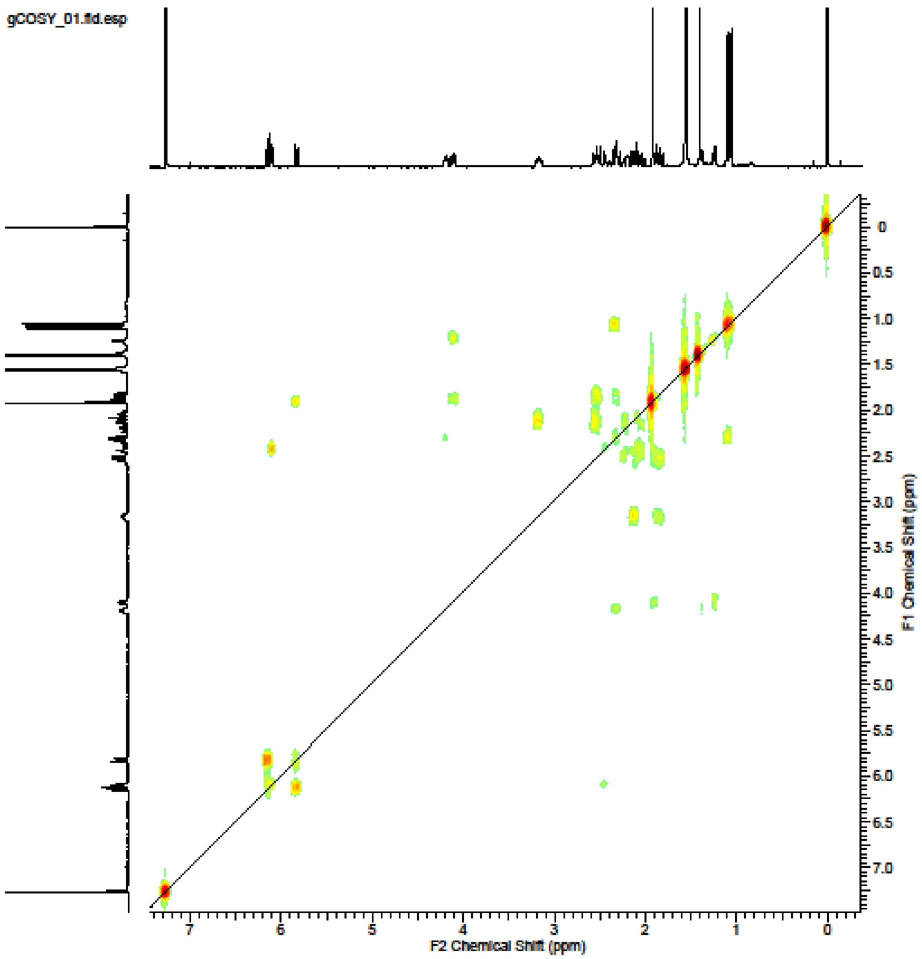

Figure S8. COSY spectrum of **1**.

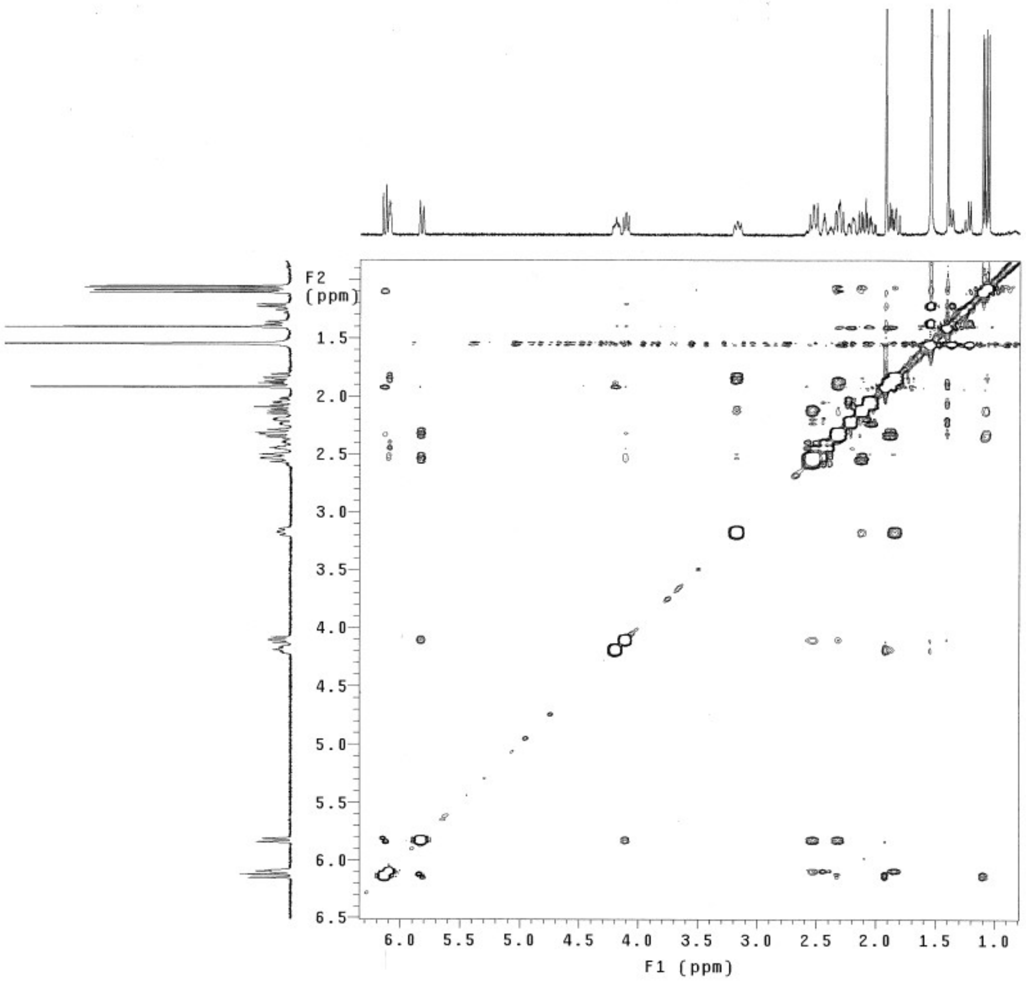

Figure S9. NOESY spectrum of **1**.

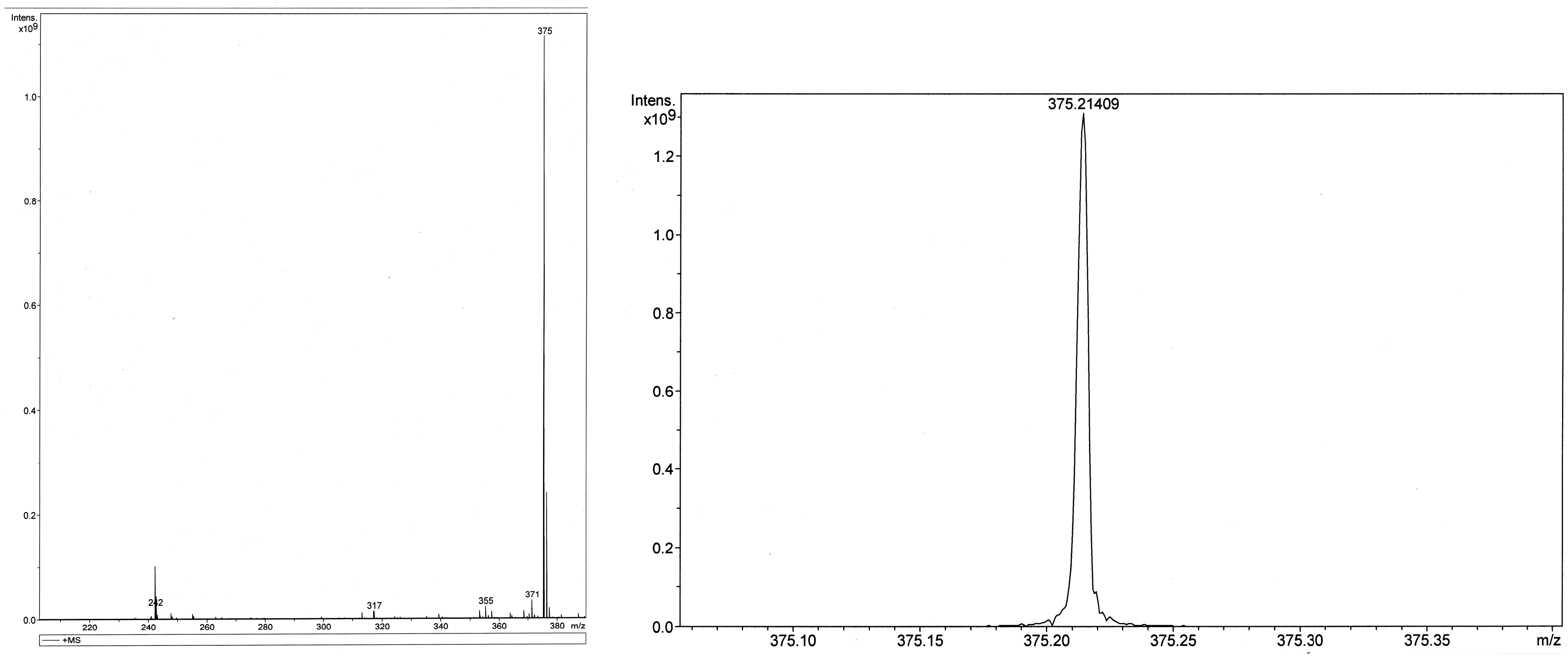

Figure S10. LR- and HR-ESIMS spectra of **2**.

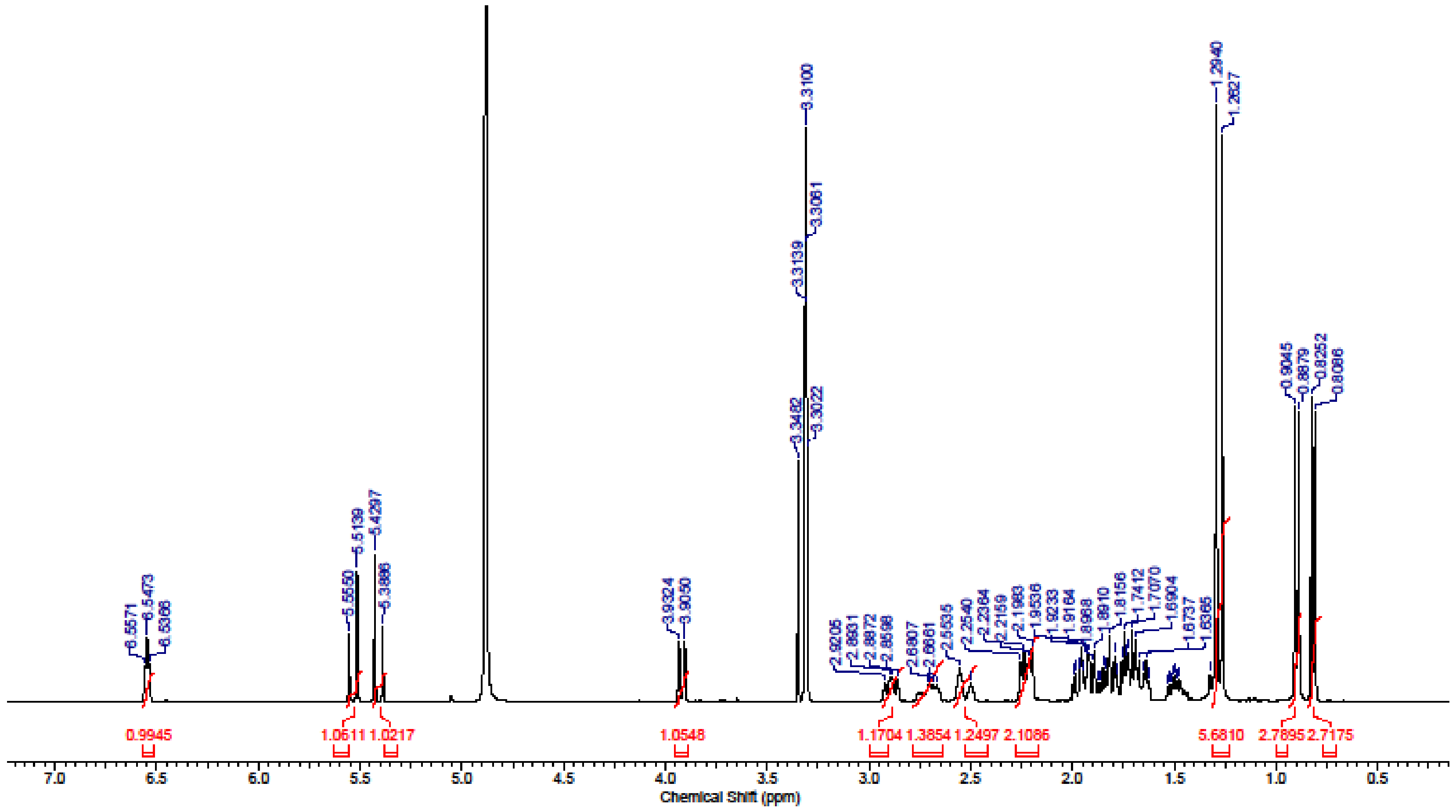

Figure S11. ^{1}H NMR spectrum of **2**.

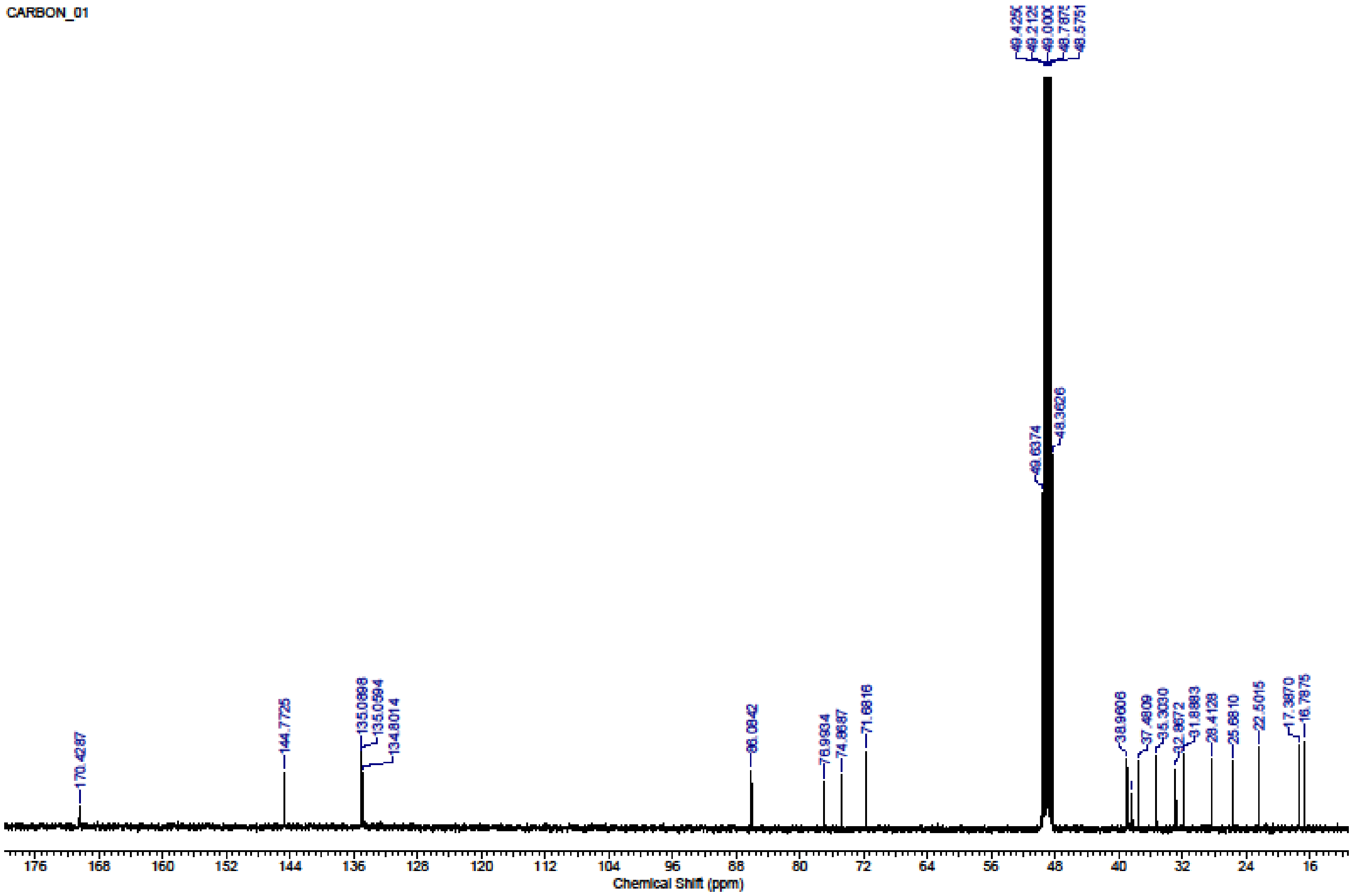

Figure S12. ^{13}C NMR spectrum of **2**.

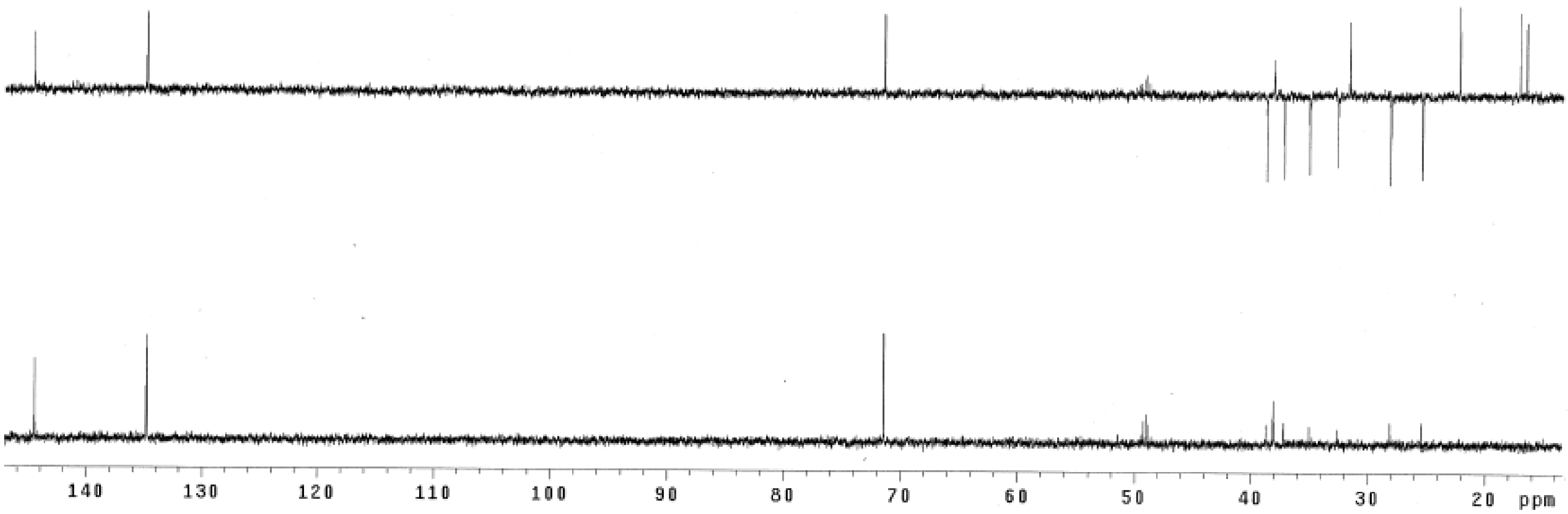

Figure S13. DEPT spectrum of **2**.

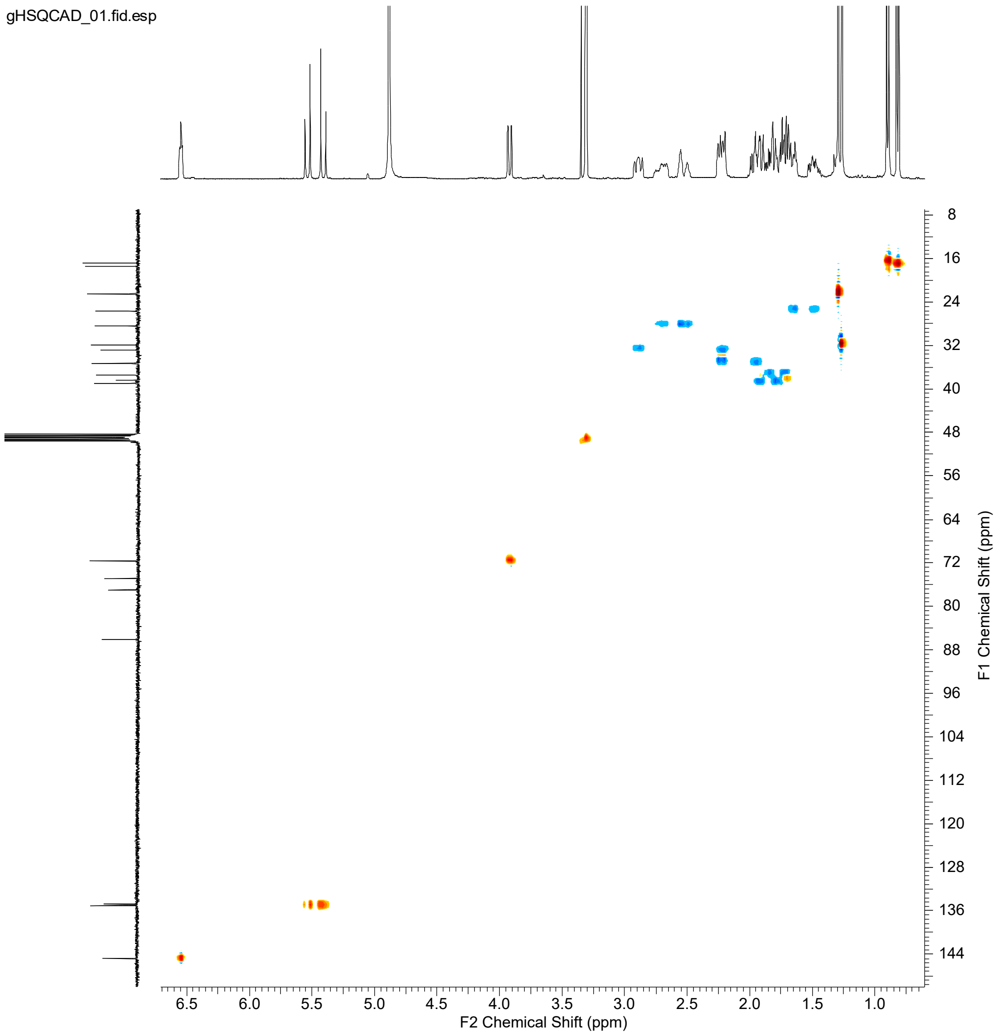

Figure S14. HSQC spectrum of **2**.

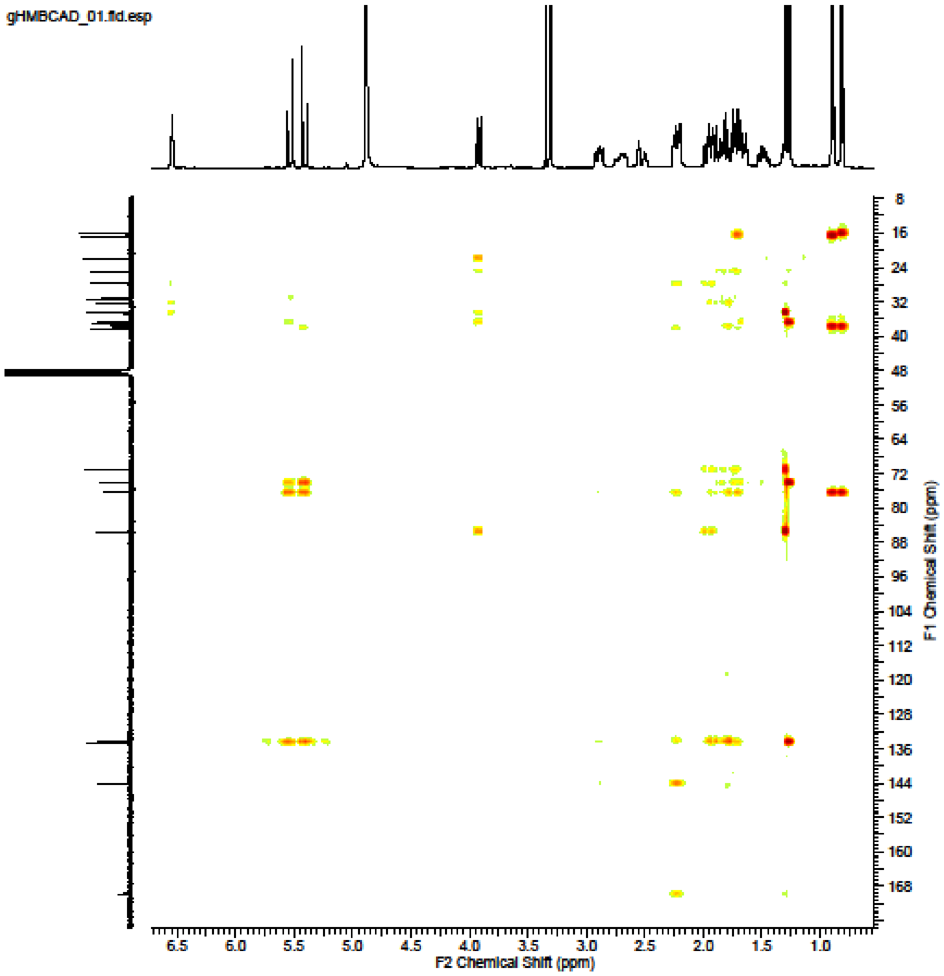

Figure S15. HMBC spectrum of **2**.

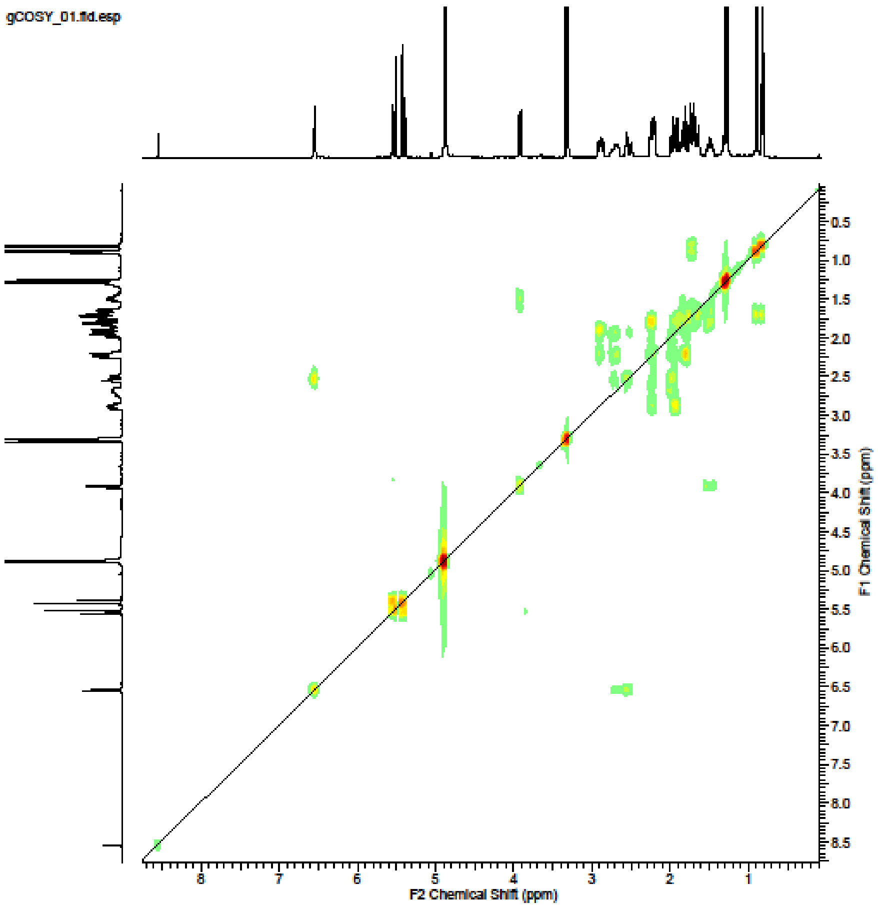

Figure S16. COSY spectrum of **2**.

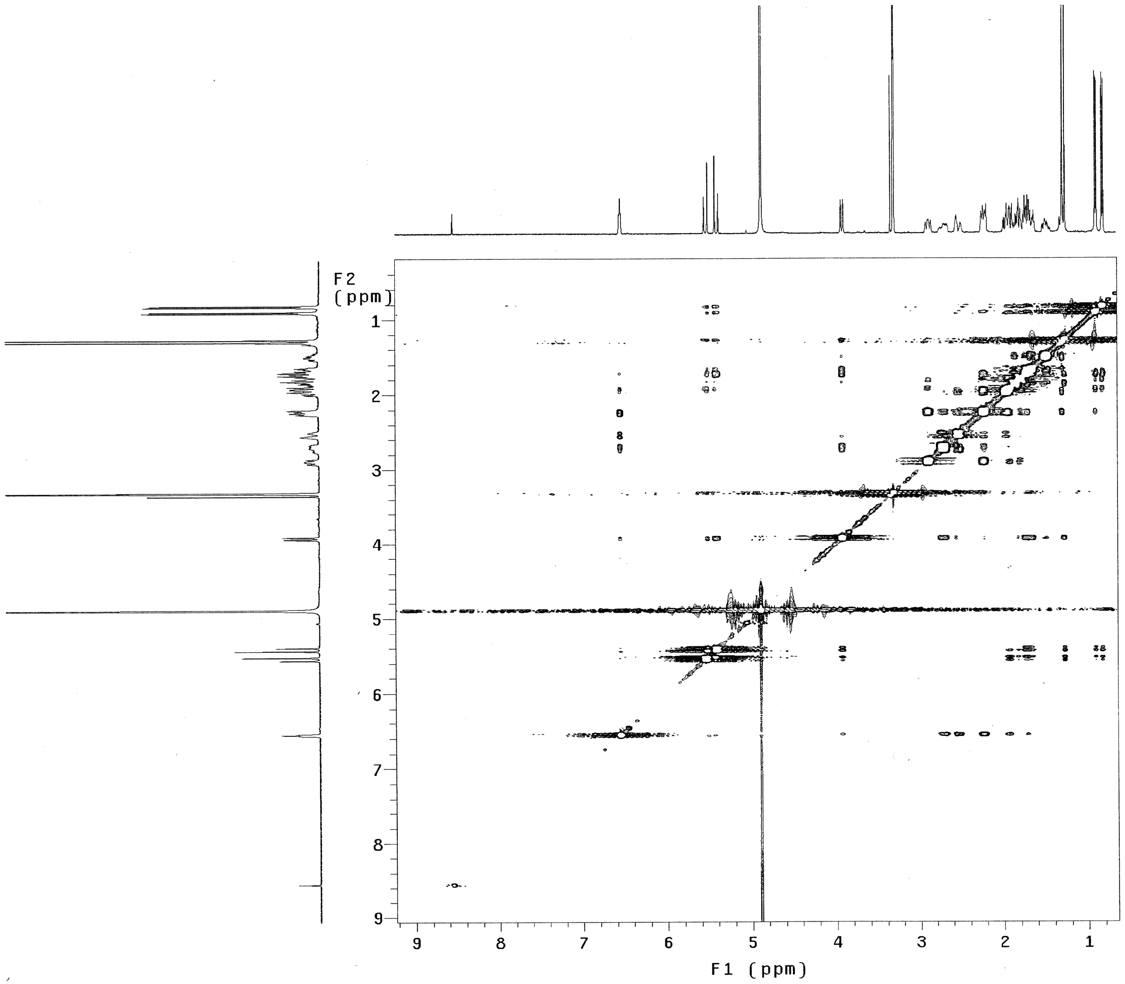

Figure S17. NOESY spectrum of **2**.

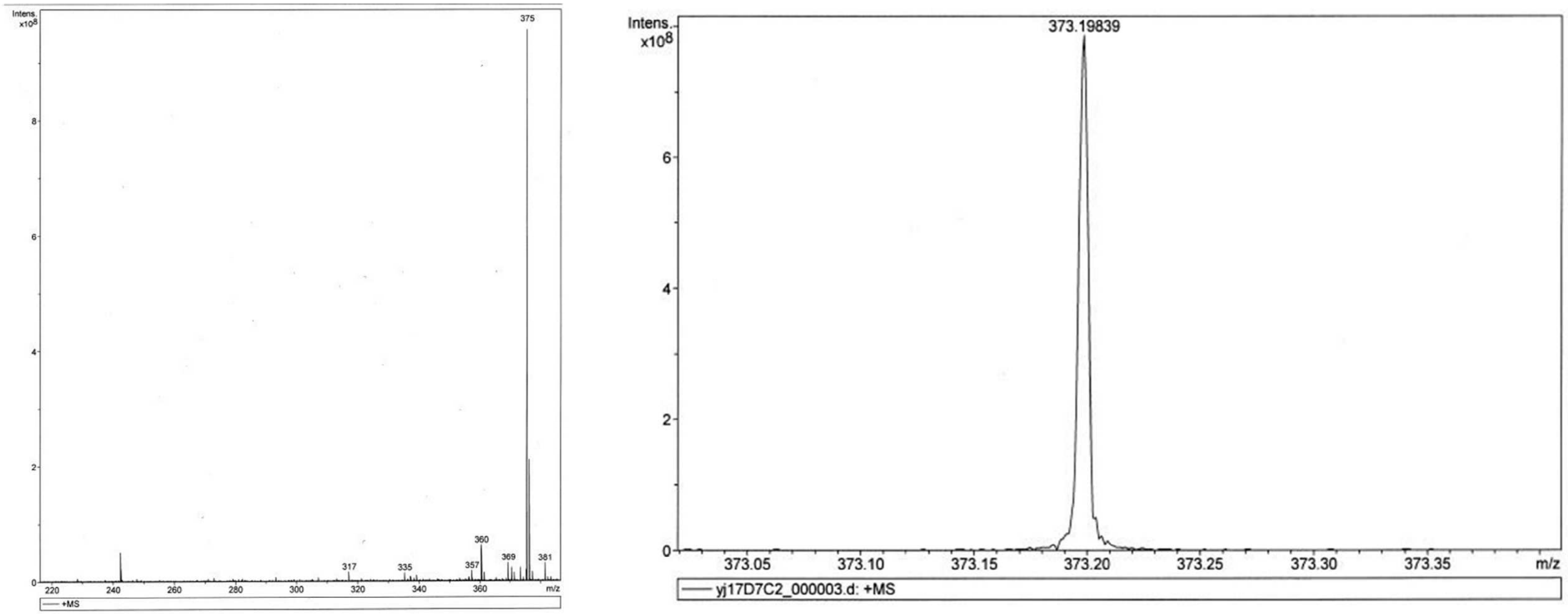

Figure S18. LR- and HR-ESIMS spectra of **3**.

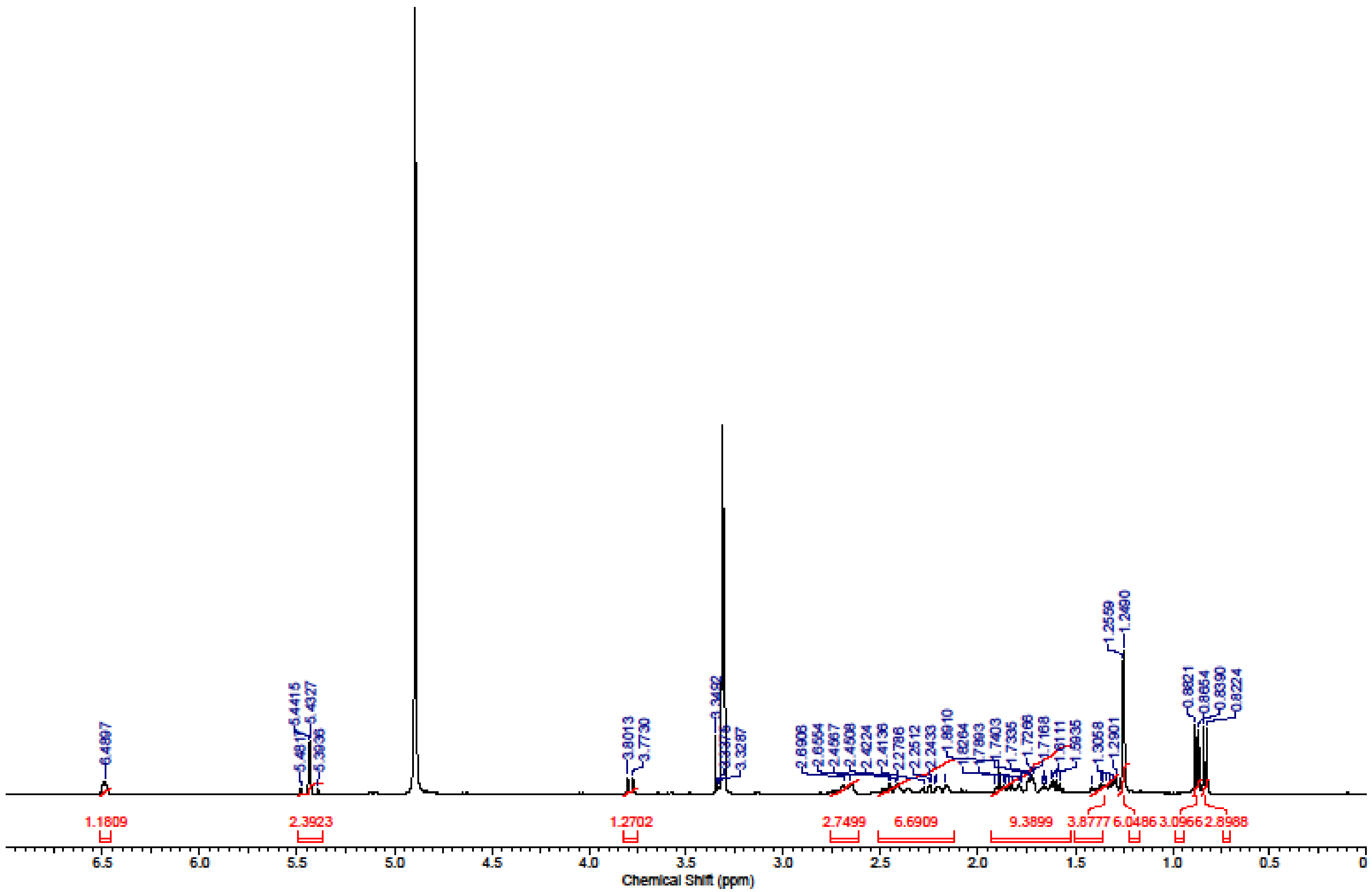

Figure S19. ^{1}H NMR spectrum of **3**.

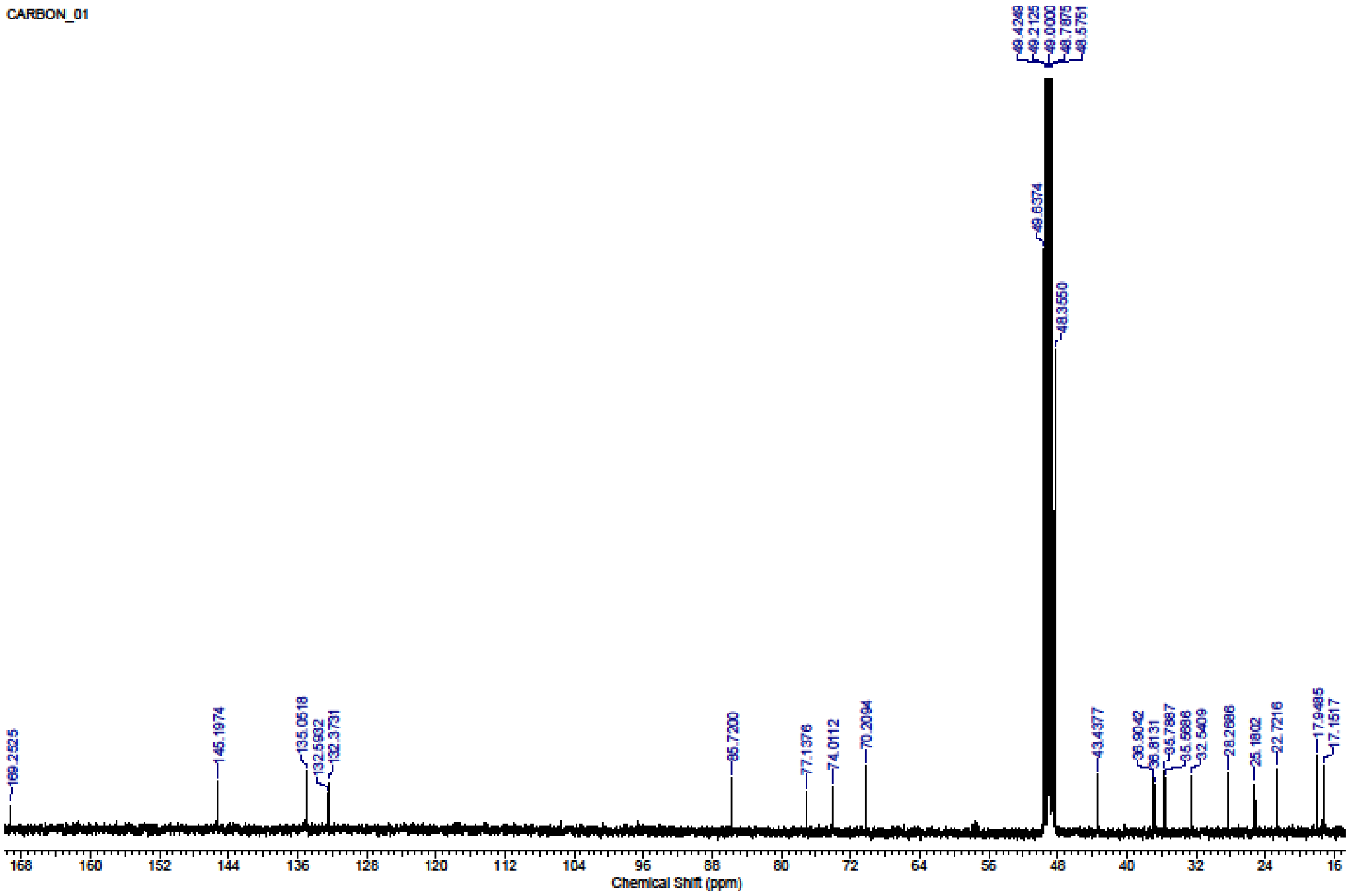

Figure S20. ^{13}C NMR spectrum of **3**.

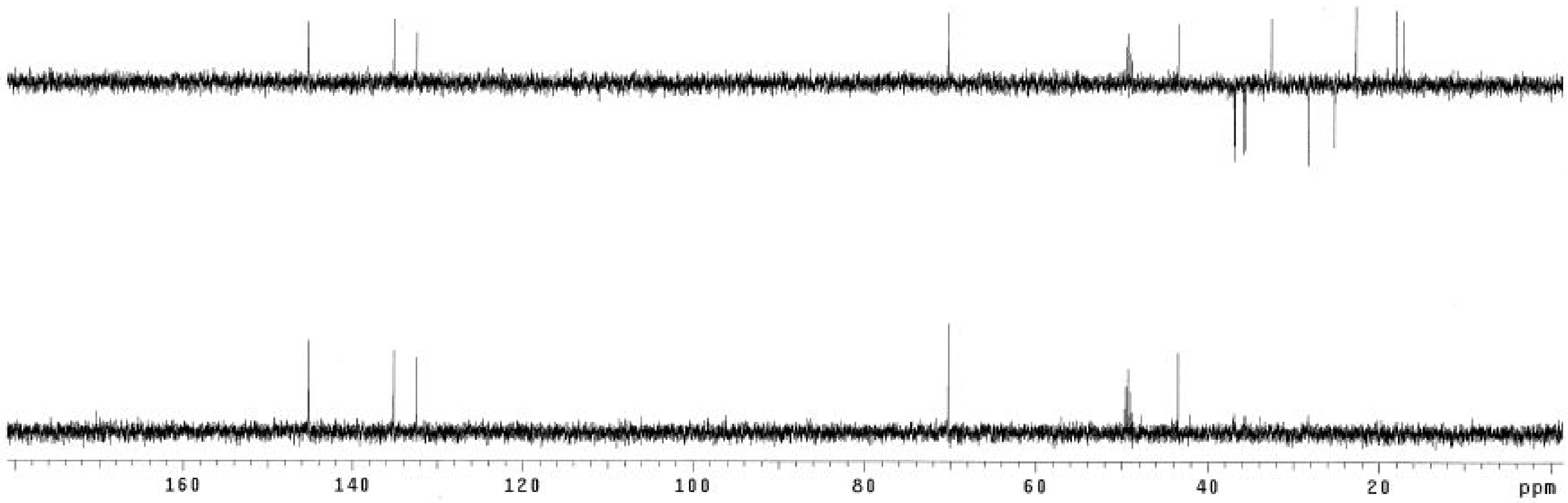

Figure S21. DEPT spectrum of **3**.

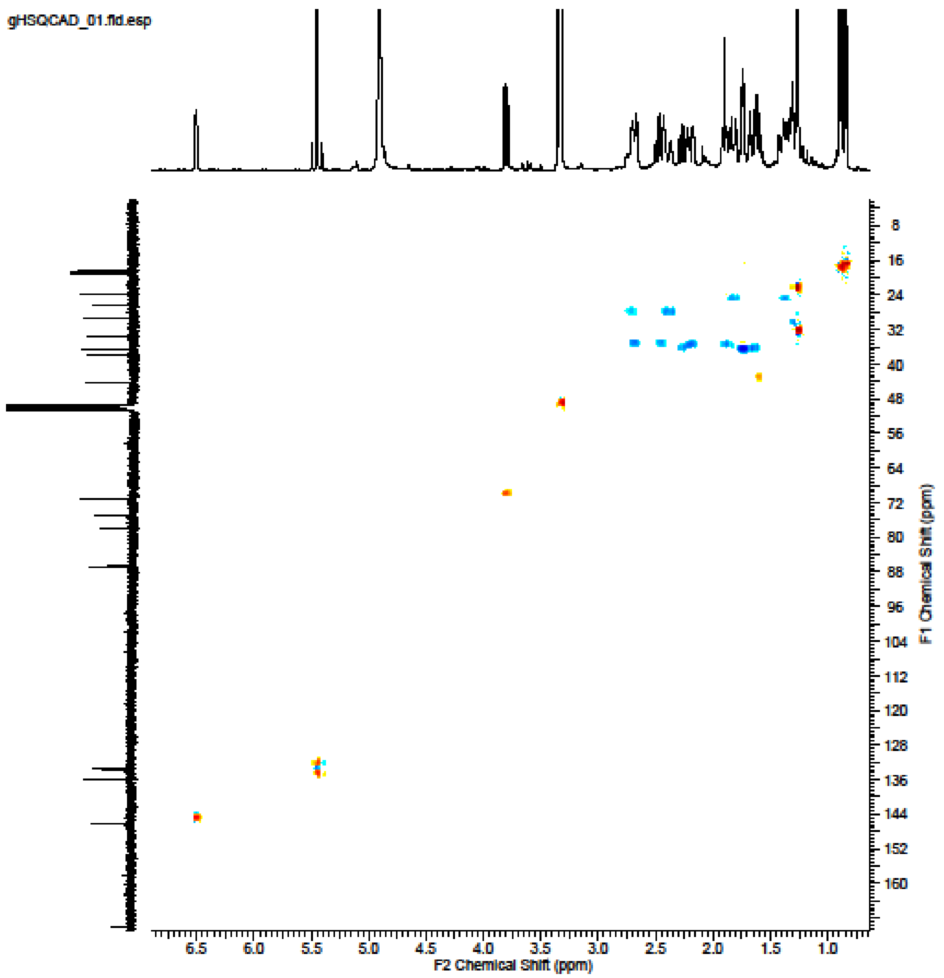

Figure S22. HSQC spectrum of **3**.

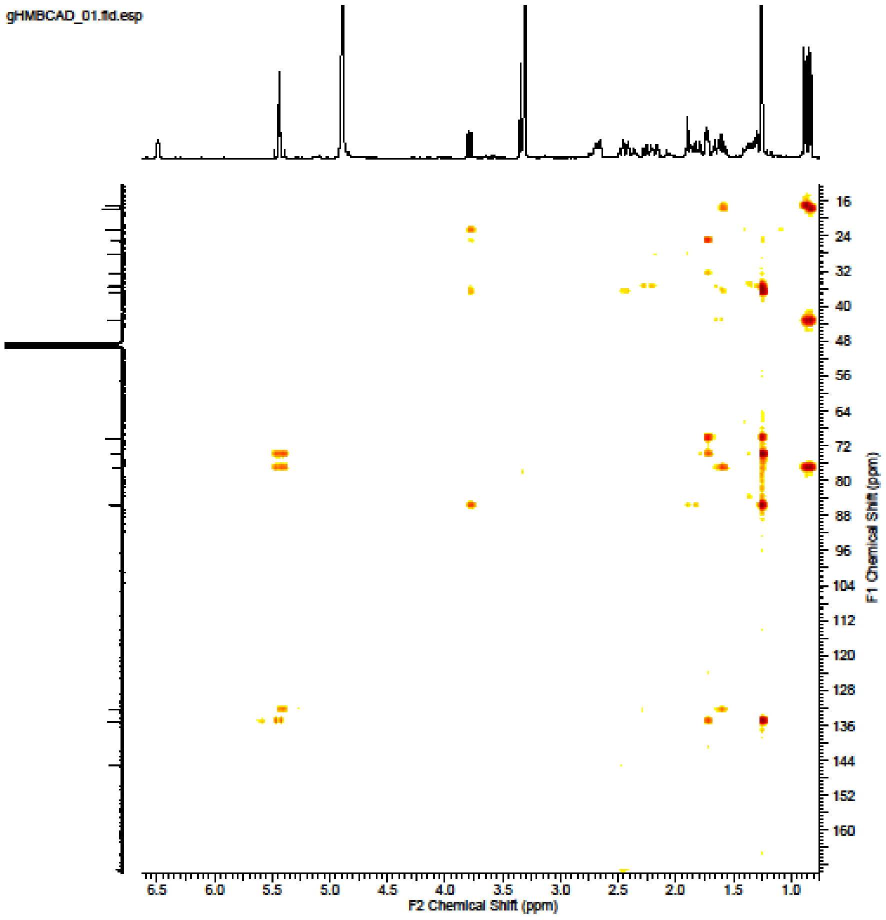

Figure S23. HMBC spectrum of **3**.

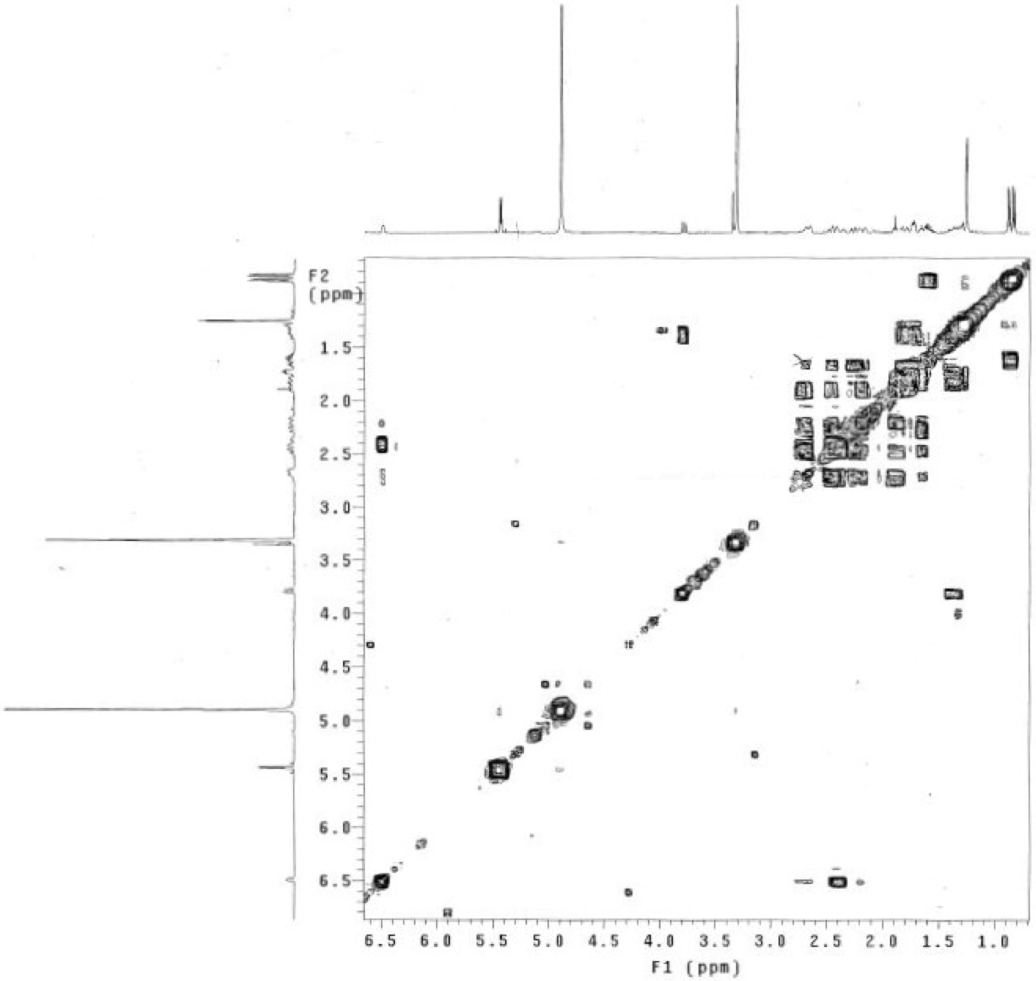

Figure S24. COSY spectrum of **3**.

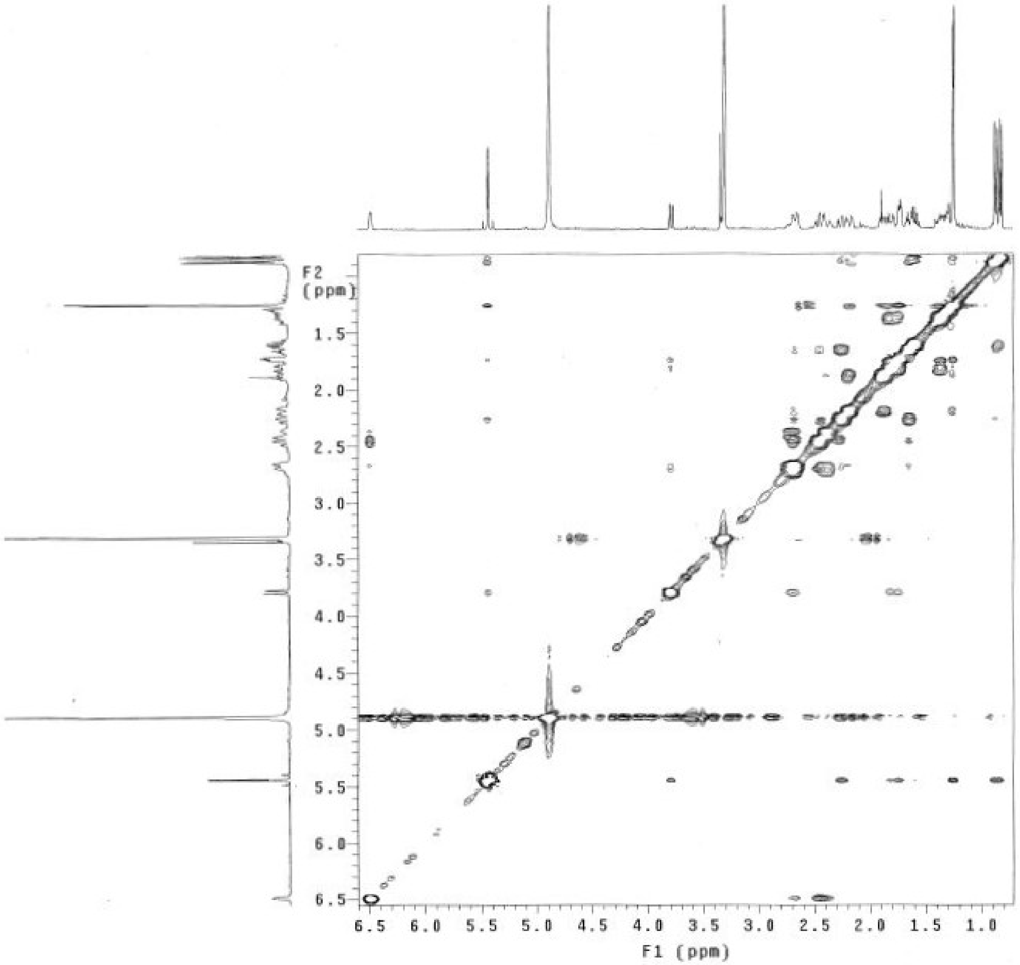

Figure S25. NOESY spectrum of **3**.

Table S1. Comparison of NMR data between **2** and sartrolide D.

	^{1}H		^{13}C	
position	**2**[a]	sartrolide D	**2**[b]	sartrolide D
1			74.5 (C)	76.6 (C)
2	5.37 d (16.4)[c]	5.56 d (16.6)[c]	134.8 (CH)[d]	134.0 (CH)
3	5.52 d (16.4)	5.39 d (16.6)	133.4 (CH)	132.9 (CH)
4			73.8 (C)	74.6 (C)
5a	1.98 m	1.97 m	34.0 (CH_2)	34.2 (CH_2)
5b	1.82 m	1.74 m		
6	1.66 m	1.54 m	25.6 (CH_2)	25.4 (CH_2)
7	3.75 d (10.0)	4.44 dd (10.8, 3.5)	72.2 (CH)	72.7 (CH)
8			83.9 (C)	83.1 (C)
9a	2.23 m	2.22 m	36.6 (CH_2)	34.7 (CH_2)
9b	2.00 m	2.01 m		
10a	2.53 m	2.57 m	27.6 (CH_2)	27.6 (CH_2)
10b	2.50 m	2.43 m		
11	6.51 t (5)	6.41 t (4.1)	144.0 (CH)	141.4 (CH)
12			134.2 (C)	133.6 (C)
13a	3.09 m	2.86 m	32.0 (CH_2)	32.4 (CH_2)
13b	2.50 m	2.51 m		
14a	1.90 m	1.89 m	38.0 (CH_2)	37.5 (CH_2)
14b	1.81 m	1.81 m		
15	1.80 m	1.78 m	40.1 (CH)	38.5 (CH)
16	0.80 d (6.8)	0.87 d (6.8)	16.7 (CH_3)	16.8 (CH_3)
17	0.85d (6.8)	0.90 d (6.8)	16.9 (CH_3)	17.4 (CH_3)
18	1.30 s	1.31 s	32.0 (CH_3)	33.1 (CH_3)
19	1.31 s	1.32 s	21.7 (CH_3)	21.7 (CH_3)
20			168.7 (C)	167.1 (C)

[a] Spectra recorded at 500 MHz in $CDCl_3$.

[b] Spectra recorded at 100 MHz in $CDCl_3$.

[c] *J* values (in Hz) in parentheses.

[d] Attached protons were deduced by DEPT experiments.

Table S2. DP4+ analysis table for compound **3** (isomer 1: 1α4β-**3**; isomer 2: 1β4α-**3**; isomer 3: 1α4α-**3**; isomer 4: 1β4β-**3**).

	A	B	C	D	E	F	G	H	I	J	K
1		Settings			Type of data (shifts)			TMS 1H	31.560	TMS 13C	196.609
2		Default			Shielding tensors			Default	μ	σ	ν
3								13Cu,sp2	-0.920	1.748	5.364
4								13Cu,sp3	2.909	1.600	6.269
5	Functional		Solvent?		Basis Set			1Hu,sp2	0.347	0.118	4.911
6	mPW1PW91		PCM		6-31+G(d,p)			1Hu,sp3	-0.018	0.112	3.651
7								13Cs	-	1.557	6.227
8								1Hs	-	0.104	3.893
9		Isomer Nº		1	2	3	4	5	6	7	8
10	DP4+ (%)		H data	100.00%	0.00%	0.00%	0.00%	-	-	-	-
11			C data	100.00%	0.00%	0.00%	0.00%	-	-	-	-
12			All data	100.00%	0.00%	0.00%	0.00%	-	-	-	-
13	Type	sp2?	Exp	1	2	3	4	5	6	7	8
14	H	x	5.44	25.7084	25.9003	25.7253	25.9193				
15	H	x	5.43	25.3996	25.4056	25.701	25.7719				
16	H		1.74	29.6438	29.7369	29.601	29.7775				
17	H		1.74	29.8941	29.8252	29.7413	29.7905				
18	H		1.82	29.6032	29.8839	30.04	29.6797				
19	H		1.37	30.2003	30.0283	30.0918	30.3644				
20	H		3.79	27.8674	27.6199	26.9805	28.0199				
21	H		2.2	29.4315	29.7114	29.7087	29.7496				
22	H		1.89	29.6998	29.3089	29.4321	29.3797				
23	H		2.67	29.1159	28.8911	29.0569	28.9721				
24	H		2.45	28.7234	28.7933	28.7765	28.6751				
25	H	x	6.49	24.67	24.3575	24.6541	24.4636				
26	H		2.72	28.748	28.7583	28.7269	28.5609				
27	H		2.38	29.1725	29.3492	29.0084	29.4427				
28	H		2.27	29.1655	29.9086	29.7937	29.7853				
[illegible]	[illegible]		[illegible]	[illegible]	[illegible]	[illegible]	[illegible]				

Main | Unscaled | Scaled | UnscaledErrors | ScaledErrors | Detailed Results | ParametersEstimati

Table S3. Conformers and Boltzmann populations of 1α4β-**3**.

1α4β-**3a** (77.42%)	1α4β-**3b** (5.85%)	1α4β-**3c** (8.60%)
C 2.29845 -0.99679 -0.25609	C 2.327191 -0.98196 -0.28065	C 2.659039 -0.5951 -0.25563
C 3.864988 -0.97178 -0.19503	C 3.893207 -0.93415 -0.11696	C 4.115967 -0.21449 0.192898
C 4.411762 -0.84589 1.235718	C 4.3639 -0.8582 1.344544	C 4.305839 -0.15357 1.717396
C 4.486001 0.097133 -1.10372	C 4.548845 0.176278 -0.94914	C 4.60545 1.086807 -0.45878
C 1.724334 0.298536 0.297493	C 1.71965 0.298678 0.250324	C 1.668406 0.459376 0.186559
C 1.775316 -2.23026 0.548964	C 1.7781 -2.23218 0.473047	C 2.27774 -1.98227 0.332876
O 2.00165 -1.14247 -1.65207	O 2.023411 -1.05861 -1.6827	O 2.62201 -0.64957 -1.69162
C 1.324536 1.347002 -0.42992	C 1.288565 1.333414 -0.47711	C 1.084365 1.363372 -0.60231
C 0.913675 2.699279 0.129128	C 0.889787 2.68604 0.091931	C 0.081582 2.410135 -0.14715
C 0.545782 -2.96357 -0.02486	C 0.545404 -2.9315 -0.13135	C 1.132254 -2.7528 -0.35532
C -0.76094 -2.19016 -0.1574	C -0.76701 -2.16516 -0.2144	C -0.2673 -2.14973 -0.37892
C -1.29298 -1.70871 1.166698	C -1.28218 -1.73977 1.12968	C -0.74892 -1.62504 0.935735
O -2.22414 -0.73219 1.312611	O -2.22238 -0.77708 1.320144	O -1.5533 -0.50645 0.956014
C -3.01807 -0.05115 0.29991	C -3.03297 -0.07586 0.335746	C -2.92397 -0.39373 0.428355
C -3.61788 -1.05966 -0.69201	C -3.64427 -1.06502 -0.66906	C -3.28652 -1.60087 -0.45167
C -2.68242 -1.55766 -1.78907	C -2.72074 -1.5162 -1.79588	C -2.46154 -1.89634 -1.70727
C -1.37036 -2.13819 -1.35912	C -1.3881 -2.07405 -1.4051	C -1.01223 -2.22496 -1.49579
O -0.85304 -2.18969 2.192785	O -0.81951 -2.24127 2.137402	O -0.30894 -2.03672 1.986711
C -2.13413 1.02614 -0.39377	C -2.16735 1.017702 -0.35509	C -3.03409 0.985499 -0.30624
C -1.59321 2.106248 0.547981	C -1.59701 2.075853 0.59339	C -1.91279 1.245401 -1.37123
C -0.57114 3.044437 -0.12381	C -0.60379 3.028479 -0.10048	C -1.07175 2.522336 -1.17819
C 1.797851 3.784021 -0.52166	C 1.743663 3.771629 -0.59742	C 0.772769 3.773805 0.004013
O 1.069622 2.754117 1.552337	O 1.102988 2.749178 1.508376	O -0.44441 2.103308 1.160904
O -2.84661 1.600276 -1.498	O -2.9151 1.621697 -1.42101	O -3.18664 2.059769 0.604446
C -4.15369 0.568738 1.124246	C -4.1555 0.523321 1.192723	C -3.83253 -0.40591 1.664918
H 4.166508 -1.95282 -0.58959	H 4.242996 -1.90077 -0.51961	H 4.75137 -1.03632 -0.18137
H 5.503271 -0.94393 1.228357	H 3.95074 -1.66135 1.962308	H 4.039439 -1.09187 2.213331
H 4.016314 -1.61317 1.909131	H 4.083379 0.097663 1.802426	H 3.704022 0.647964 2.160892
H 4.177189 0.134646 1.667749	H 5.456418 -0.9323 1.391202	H 5.354957 0.057809 1.953902
H 4.251884 1.108486 -0.75207	H 4.303423 1.167141 -0.55195	H 4.056622 1.951651 -0.07103
H 4.124257 0.000802 -2.13001	H 4.216977 0.145883 -1.99063	H 4.47142 1.066985 -1.54403
H 5.57753 -0.00806 -1.11028	H 5.639635 0.068219 -0.93025	H 5.66964 1.239504 -0.24487
H 1.686556 0.369549 1.383245	H 1.703942 0.377894 1.335573	H 1.447906 0.458014 1.252236
H 2.574648 -2.98108 0.58453	H 2.570895 -2.99349 0.50159	H 3.163451 -2.63183 0.26864
H 1.569993 -1.94504 1.584287	H 1.566726 -1.97635 1.5147	H 2.052293 -1.88142 1.3976

H 1.036178 -1.08407 -1.73819
H 1.360022 1.28131 -1.51718
H 0.806653 -3.36747 -1.00798
H 0.358513 -3.81791 0.635671
H -3.998 -1.90378 -0.10256
H -4.48342 -0.59307 -1.17264
H -2.48648 -0.74147 -2.49916
H -3.20734 -2.32303 -2.37956
H -0.83199 -2.62036 -2.17766
H -1.28663 0.512652 -0.85328
H -2.44 2.706226 0.910311
H -1.14949 1.642653 1.433828
H -0.71371 4.062969 0.255838
H -0.75493 3.082676 -1.20399
H 2.857713 3.593034 -0.3135
H 1.537452 4.768147 -0.11817
H 1.672098 3.806265 -1.61007
H 1.998098 2.547948 1.747925
H -3.45917 2.262754 -1.14003
H -3.76133 1.134268 1.972389
H -4.80432 -0.21928 1.51582
H -4.76517 1.233341 0.503403

H 2.561275 -1.77704 -2.05244
H 1.284965 1.254164 -1.56261
H 0.791816 -3.28457 -1.13746
H 0.37027 -3.82192 0.484891
H -3.99957 -1.93005 -0.09447
H -4.52641 -0.59817 -1.11855
H -2.55552 -0.67945 -2.48943
H -3.24008 -2.27929 -2.39504
H -0.8358 -2.48389 -2.25092
H -1.33463 0.517456 -0.85415
H -2.43005 2.666052 1.001116
H -1.11925 1.5932 1.45094
H -0.73445 4.041592 0.297746
H -0.82804 3.079807 -1.17226
H 2.811789 3.587688 -0.42781
H 1.493449 4.756955 -0.19024
H 1.577197 3.786475 -1.68044
H 2.039009 2.544886 1.66618
H -3.52622 2.262395 -1.0231
H -3.74948 1.075393 2.043528
H -4.79417 -0.27547 1.582373
H -4.78292 1.195667 0.5966

H 3.37581 -1.19216 -1.97405
H 1.321696 1.366629 -1.66407
H 1.414718 -2.96245 -1.39151
H 1.066494 -3.72125 0.159533
H -3.26518 -2.49252 0.189913
H -4.33502 -1.47207 -0.74644
H -2.54713 -1.08336 -2.44006
H -2.9193 -2.7645 -2.20537
H -0.52099 -2.63868 -2.37692
H -3.99894 0.942318 -0.82692
H -1.22742 0.396019 -1.41988
H -2.39446 1.313869 -2.35377
H -1.71859 3.358465 -0.8931
H -0.62446 2.78382 -2.14586
H 1.565064 3.707946 0.755127
H 0.047971 4.529864 0.325964
H 1.214307 4.097146 -0.94465
H -0.62276 1.141867 1.19828
H -2.30357 2.256786 0.985604
H -3.55657 0.398601 2.346038
H -3.74515 -1.36469 2.186092
H -4.87817 -0.25636 1.373564

1α4β-**3d** (5.23%)

C -2.56178 -0.53948 0.5285
C -3.85524 -0.87769 -0.28463
C -4.80464 0.331809 -0.35843
C -4.60741 -2.09856 0.266032
C -1.82131 0.602545 -0.1472
C -1.62104 -1.75834 0.700643
O -2.9017 -0.20386 1.884923
C -1.15362 1.573859 0.481498
C -0.44492 2.725883 -0.21666
C -0.96559 -2.34509 -0.58793
C 0.541178 -2.16042 -0.61322
C 1.059529 -0.96574 -1.33319
O 2.367793 -0.61085 -1.19877
C 3.038144 -0.34642 0.07881

1α4β-**3e** (2.90%)

C 2.695519 -0.17633 -0.25839
C 4.071574 0.443478 0.188795
C 5.283423 -0.35453 -0.32239
C 4.195139 0.688051 1.701759
C 1.575136 0.764013 0.159437
C 2.481491 -1.58168 0.349555
O 2.718948 -0.35213 -1.68237
C 0.83769 1.512804 -0.66267
C -0.27012 2.463405 -0.23531
C 1.433448 -2.49681 -0.31749
C -0.03196 -2.07848 -0.3387
C -0.56737 -1.58389 0.966415
O -1.5065 -0.57319 0.961093
C -2.88752 -0.65971 0.458117

C	3.161165	-1.61883	0.95808
C	2.852854	-2.90698	0.171274
C	1.368928	-3.05279	-0.03659
O	0.377101	-0.34665	-2.13449
C	2.215669	0.761123	0.788899
C	2.108952	2.068911	-0.01116
C	0.963751	3.015643	0.395438
C	-1.30766	3.991988	-0.05258
O	-0.34601	2.527396	-1.62276
O	2.851978	0.965401	2.052992
C	4.427006	0.132551	-0.34464
H	-3.53273	-1.10306	-1.31126
H	-5.64939	0.115365	-1.02137
H	-4.30315	1.228938	-0.73679
H	-5.22465	0.56524	0.628808
H	-4.81271	-1.97201	1.334637
H	-4.04653	-3.02986	0.13566
H	-5.56534	-2.22118	-0.25229
H	-1.8319	0.597246	-1.23359
H	-0.84106	-1.43844	1.398694
H	-2.18141	-2.5414	1.220489
H	-3.35792	0.652565	1.857122
H	-1.13963	1.594636	1.571921
H	-1.18308	-3.41736	-0.64045
H	-1.39179	-1.89564	-1.48781
H	4.176964	-1.66541	1.364585
H	2.490864	-1.55186	1.820067
H	3.224031	-3.77285	0.72876
H	3.386482	-2.89333	-0.78801
H	0.908275	-3.94152	0.395413
H	1.208151	0.34633	0.952498
H	3.06114	2.598851	0.112534
H	2.01627	1.8502	-1.0788
H	1.242021	4.023394	0.065679
H	0.871257	3.074827	1.491816
H	-2.30372	3.818323	-0.46978
H	-0.84618	4.827718	-0.58962
H	-1.41125	4.268122	1.002907

C	-3.09872	-1.93098	-0.38089
C	-2.25617	-2.15692	-1.63967
C	-0.77303	-2.28314	-1.44207
O	-0.07594	-1.90707	2.025014
C	-3.18899	0.669362	-0.31359
C	-2.12659	1.040317	-1.40519
C	-1.43917	2.410901	-1.25214
C	0.269374	3.899078	-0.14354
O	-0.7432	2.149801	1.09075
O	-3.46821	1.743716	0.566802
C	-3.76933	-0.75403	1.710421
H	4.096914	1.431093	-0.30127
H	6.196688	0.240863	-0.20806
H	5.173839	-0.61964	-1.3775
H	5.429737	-1.28083	0.244991
H	5.162379	1.152437	1.925827
H	4.146662	-0.25093	2.265268
H	3.41571	1.353911	2.084053
H	1.39301	0.826405	1.230588
H	3.428926	-2.12827	0.291488
H	2.245723	-1.47513	1.411599
H	3.005757	0.489678	-2.07231
H	1.004424	1.449453	-1.73747
H	1.739549	-2.68853	-1.3498
H	1.491429	-3.45577	0.216121
H	-2.9533	-2.79129	0.286778
H	-4.15839	-1.94994	-0.66305
H	-2.45953	-1.38932	-2.39788
H	-2.60011	-3.09525	-2.10084
H	-0.2411	-2.65562	-2.31799
H	-4.14693	0.484474	-0.81543
H	-1.34706	0.276594	-1.45277
H	-2.63082	1.032693	-2.37877
H	-2.17666	3.17258	-0.97904
H	-1.0366	2.699051	-2.23219
H	0.6583	4.235642	-1.11072
H	1.075519	3.946865	0.593984
H	-0.52929	4.58232	0.165959

H	-0.00288	1.631365	-1.80473	H	-0.80328	1.175604	1.166996
H	2.361265	1.659285	2.519683	H	-2.61437	2.061771	0.931773
H	4.361998	0.932898	-1.08546	H	-3.59522	0.101586	2.362361
H	4.991744	-0.69528	-0.78668	H	-3.5461	-1.67505	2.258419
H	4.96783	0.49861	0.532571	H	-4.82958	-0.75598	1.433764

Table S4. Conformer and Boltzmann population of 1β4α-**3**.

1β4α-**3a** (100%)

C	-2.44679	-0.86718	-0.20796
C	-3.97219	-0.61058	0.068856
C	-4.5296	0.571647	-0.73517
C	-4.31524	-0.45252	1.558577
C	-1.61	0.3014	0.306707
C	-2.03459	-2.19583	0.495357
O	-2.36283	-1.00156	-1.62152
C	-1.22682	1.357991	-0.4157
C	-0.48611	2.577751	0.106744
C	-0.77666	-2.93137	-0.0198
C	0.559399	-2.21277	0.162294
C	1.097341	-1.59734	-1.09311
O	2.355452	-1.11763	-1.20864
C	3.066314	-0.30589	-0.22019
C	3.461384	-1.16317	0.986974
C	2.337138	-1.53127	1.950819
C	1.112812	-2.19267	1.391724
O	0.418874	-1.59163	-2.11301
C	2.130868	0.873599	0.144922
C	1.719681	1.73135	-1.06229
C	0.778291	2.900059	-0.72177
C	-1.43137	3.794887	0.088872
O	-0.01424	2.390525	1.462176
O	2.771202	1.64422	1.159776
C	4.332489	0.141766	-0.95258
H	-4.46984	-1.51772	-0.30198
H	-4.30597	0.461033	-1.79845
H	-5.61822	0.624202	-0.61294
H	-4.11119	1.524812	-0.39185
H	-5.39826	-0.33785	1.682062

H	-4.00791	-1.3148	2.159438
H	-3.84458	0.442682	1.983988
H	-1.34425	0.256432	1.362509
H	-1.91974	-2.01467	1.571811
H	-2.86989	-2.89924	0.385277
H	-1.43428	-1.19517	-1.8631
H	-1.49165	1.390911	-1.47182
H	-0.72454	-3.8814	0.525737
H	-0.91388	-3.18514	-1.07219
H	3.929086	-2.07419	0.593838
H	4.228343	-0.62266	1.550484
H	2.013096	-0.62679	2.488909
H	2.74702	-2.18899	2.729464
H	0.530825	-2.71049	2.157412
H	1.220631	0.432052	0.560358
H	1.249648	1.084526	-1.81058
H	2.614783	2.158167	-1.52883
H	1.325866	3.674139	-0.17038
H	0.457189	3.360009	-1.66423
H	-1.83746	3.968095	-0.91333
H	-2.27948	3.631337	0.765179
H	-0.89566	4.694875	0.410318
H	-0.78787	2.262633	2.033874
H	2.044742	2.119612	1.602259
H	4.094257	0.546079	-1.93878
H	4.840419	0.911832	-0.36445
H	5.013033	-0.70494	-1.08996

Table S5. Conformers and Boltzmann populations of 1α4α-**3**.

1α4α-**3a** (27.46%)	1α4α-**3b** (13.96%)	1α4α-**3c** (34.39%)
C -2.57091 -0.66625 0.232318	C -2.47701 -0.83469 0.292251	C -2.57129 -0.67029 0.244721
C -3.81864 -0.18714 -0.59337	C -3.92162 -0.76922 -0.32883	C -3.77774 -0.22526 -0.65458
C -4.59055 0.923643 0.140543	C -3.96014 -0.93196 -1.85733	C -4.61454 0.883529 0.013876
C -4.80175 -1.31707 -0.95693	C -4.68679 0.492448 0.095609	C -4.71594 -1.38253 -1.04104
C -1.58863 0.471869 0.480945	C -1.65095 0.339977 -0.1885	C -1.57496 0.464419 0.480085
C -1.87539 -1.87916 -0.45218	C -1.82254 -2.19848 -0.07471	C -1.87739 -1.92435 -0.34482
O -2.99395 -1.04338 1.561184	O -2.59489 -0.71624 1.719395	O -3.06468 -1.09639 1.532934
C -1.30377 1.492174 -0.33285	C -1.27712 1.373699 0.567927	C -1.34509 1.535789 -0.28674

C	-0.3939	2.667907	-0.01675
C	-0.81913	-2.64858	0.376491
C	0.586618	-2.07315	0.446024
C	1.266957	-2.05298	-0.89711
O	2.358135	-1.3055	-1.19118
C	3.072813	-0.36789	-0.3309
C	3.525291	-1.08225	0.952779
C	2.463664	-1.23558	2.039232
C	1.125091	-1.78348	1.64567
O	0.81913	-2.73478	-1.80092
C	2.159277	0.849547	-0.05159
C	1.61436	1.539733	-1.31105
C	0.778822	2.798168	-1.01724
C	-1.22654	3.965943	-0.04211
O	0.210975	2.54922	1.288541
O	2.901741	1.751197	0.771783
C	4.304949	0.006334	-1.15907
H	-3.43692	0.219098	-1.54027
H	-5.42027	1.282843	-0.47886
H	-3.95326	1.777767	0.381448
H	-5.00454	0.543712	1.080163
H	-5.14914	-1.858	-0.06573
H	-5.69327	-0.89074	-1.42977
H	-4.3829	-2.04633	-1.6555
H	-1.09087	0.402214	1.445791
H	-2.65486	-2.61158	-0.69152
H	-1.45258	-1.56751	-1.41268
H	-3.55931	-1.82728	1.471052
H	-1.79154	1.569632	-1.30446
H	-1.19941	-2.77303	1.395033
H	-0.7415	-3.64569	-0.06986
H	3.909598	-2.06478	0.651192
H	4.366977	-0.52648	1.377435
H	2.2904	-0.25531	2.508458
H	2.866526	-1.86502	2.84651
H	0.467447	-1.94343	2.50175
H	1.299213	0.485536	0.510744
H	1.016085	0.817149	-1.87699

C	-0.55886	2.643333	0.152664
C	-0.67228	-2.70497	0.822565
C	0.687529	-2.03194	0.695016
C	1.261748	-2.13263	-0.69028
O	2.217852	-1.30689	-1.17736
C	2.95736	-0.25532	-0.48386
C	3.582458	-0.82607	0.799541
C	2.645263	-0.92979	2.001833
C	1.304215	-1.57044	1.798635
O	0.831995	-2.97669	-1.45605
C	2.023472	0.948684	-0.20274
C	1.232143	1.43164	-1.4252
C	0.261954	2.606517	-1.15583
C	-1.58426	3.792448	0.079167
O	0.405633	2.994494	1.191007
O	2.843299	1.973106	0.354677
C	4.066467	0.10106	-1.47771
H	-4.44847	-1.63805	0.101668
H	-3.48446	-1.85734	-2.19525
H	-3.46719	-0.09259	-2.36183
H	-5.00007	-0.95032	-2.203
H	-4.65338	0.6329	1.179538
H	-4.26366	1.388883	-0.37094
H	-5.73645	0.419078	-0.21171
H	-1.40302	0.31371	-1.24677
H	-2.61453	-2.96045	-0.02325
H	-1.4876	-2.18937	-1.11528
H	-3.20941	-1.40849	2.011373
H	-1.5715	1.354472	1.618405
H	-0.98633	-2.65156	1.869114
H	-0.5384	-3.76439	0.57405
H	3.99221	-1.81188	0.545898
H	4.428069	-0.19152	1.081936
H	2.470814	0.078472	2.406994
H	3.16095	-1.46922	2.810483
H	0.731526	-1.65396	2.723528
H	1.290427	0.628412	0.54161
H	0.687257	0.578043	-1.83787

C	-0.40219	2.686726	0.022267
C	-0.80798	-2.63059	0.521663
C	0.602619	-2.06105	0.522516
C	1.239858	-2.0861	-0.84189
O	2.295398	-1.31687	-1.20753
C	3.048608	-0.36829	-0.39421
C	3.558471	-1.06547	0.877928
C	2.548828	-1.19991	2.015804
C	1.189961	-1.74448	1.691881
O	0.78266	-2.81852	-1.69938
C	2.150007	0.854197	-0.09001
C	1.555004	1.536235	-1.33147
C	0.741249	2.803513	-1.01393
C	-1.20443	4.003516	0.042725
O	0.240397	2.533582	1.305805
O	2.927286	1.76297	0.693625
C	4.242465	-0.00723	-1.28233
H	-3.35286	0.182408	-1.58181
H	-5.35456	1.276752	-0.69152
H	-4.00486	1.723429	0.359674
H	-5.17546	0.486935	0.870448
H	-5.01717	-1.94676	-0.1514
H	-5.62293	-0.98655	-1.51222
H	-4.25999	-2.07765	-1.75122
H	-1.00208	0.342158	1.398078
H	-2.66209	-2.6677	-0.50678
H	-1.46809	-1.67999	-1.32997
H	-3.47966	-0.32806	1.954948
H	-1.89245	1.666524	-1.21963
H	-1.17352	-2.68714	1.550842
H	-0.74094	-3.65672	0.143543
H	3.925629	-2.05344	0.572693
H	4.420897	-0.50682	1.25444
H	2.404138	-0.21286	2.480338
H	2.987015	-1.82212	2.810288
H	0.566867	-1.87939	2.577833
H	1.314272	0.496853	0.510989
H	0.929494	0.812264	-1.86509

H 2.442742 1.843631 -1.96134

H 1.432136 3.591883 -0.6349

H 0.369019 3.165827 -1.9661

H -0.58008 4.829805 0.14581

H -1.72258 4.105312 -1.00875

H -2.0047 3.938598 0.730552

H -0.50223 2.481623 1.943854

H 2.22366 2.268025 1.242824

H 4.022498 0.291341 -2.17548

H 4.825652 0.843109 -0.68527

H 4.987934 -0.84675 -1.22749

H 1.92645 1.759893 -2.20751

H 0.828109 3.545544 -1.16221

H -0.437 2.672052 -1.99843

H -2.12 3.893419 1.030927

H -1.07319 4.738887 -0.12849

H -2.32675 3.608242 -0.70424

H -0.02991 2.899172 2.05302

H 2.214404 2.562742 0.813487

H 3.651589 0.299535 -2.46938

H 4.600433 0.989227 -1.13124

H 4.771686 -0.73153 -1.56931

H 2.356963 1.830293 -2.01831

H 1.414824 3.590489 -0.65321

H 0.305601 3.174663 -1.94981

H -0.5337 4.848404 0.231011

H -1.72113 4.173594 -0.90801

H -1.96369 3.979804 0.834503

H -0.45357 2.470674 1.981625

H 2.270861 2.280823 1.192508

H 3.914606 0.263263 -2.28908

H 4.784921 0.835615 -0.84488

H 4.921 -0.86213 -1.36917

1α4α-**3d** (21.55%)

C -2.42647 -0.87913 0.257896

C -3.92245 -0.80503 -0.20246

C -4.09166 -0.86174 -1.729

C -4.66473 0.407702 0.375822

C -1.64342 0.321164 -0.25909

C -1.80487 -2.2236 -0.23251

O -2.49539 -0.86494 1.69097

C -1.31955 1.381679 0.485402

C -0.61394 2.663207 0.085324

C -0.65033 -2.82533 0.601234

C 0.689829 -2.10199 0.583196

C 1.318922 -2.09053 -0.78547

O 2.299397 -1.24201 -1.16855

C 2.975177 -0.20364 -0.3957

C 3.538052 -0.80551 0.90136

C 2.538259 -0.99226 2.041168

C 1.243993 -1.68875 1.739231

O 0.922104 -2.87706 -1.62553

C 2.000445 0.973536 -0.13658

C 1.267094 1.461466 -1.39434

C 0.272464 2.626754 -1.17894

C -1.65723 3.788702 -0.05382

O 0.293902 3.043252 1.163872

O 2.768323 1.99897 0.485948

C 4.134959 0.206248 -1.30847

1α4α-**3e** (2.10%)

C -2.577 -0.31048 0.259264

C -3.97454 0.045743 -0.37801

C -5.12779 -0.79586 0.196804

C -4.00376 -0.00444 -1.91481

C -1.56348 0.726077 -0.21064

C -2.14824 -1.7533 -0.10491

O -2.71032 -0.2725 1.687611

C -0.96137 1.617115 0.579388

C -0.0614 2.774628 0.184689

C -1.1049 -2.44482 0.801264

C 0.35217 -2.0197 0.681259

C 0.907423 -2.21603 -0.70176

O 1.99385 -1.56488 -1.18347

C 2.903701 -0.6643 -0.48194

C 3.415651 -1.34418 0.798666

C 2.471262 -1.2911 1.999203

C 1.037503 -1.68059 1.788854

O 0.344006 -2.97194 -1.47235

C 2.19501 0.683015 -0.19364

C 1.489193 1.298135 -1.4093

C 0.741318 2.621777 -1.1272

C -0.90059 4.066506 0.127076

O 0.948226 2.960743 1.219622

O 3.183016 1.549808 0.359334

C 4.060575 -0.50036 -1.47185

H -4.38326 -1.70667 0.225176
H -3.66974 0.027603 -2.21274
H -5.15719 -0.89322 -1.98316
H -3.62167 -1.74432 -2.17398
H -5.73028 0.345348 0.125135
H -4.57031 0.446944 1.463717
H -4.28112 1.347867 -0.03671
H -1.37158 0.284389 -1.31187
H -2.60523 -2.97398 -0.22248
H -1.47912 -2.13246 -1.2726
H -1.58016 -0.86687 2.014323
H -1.65226 1.376689 1.526024
H -0.9804 -2.94073 1.6396
H -0.48674 -3.83442 0.208356
H 3.997124 -1.7661 0.636154
H 4.341691 -0.15503 1.259743
H 2.293103 -0.0079 2.468608
H 3.031845 -1.5358 2.860257
H 0.652355 -1.88007 2.637869
H 1.235009 0.62083 0.560805
H 0.751553 0.606456 -1.84022
H 1.998528 1.798289 -2.13792
H 0.826221 3.572807 -1.16311
H -0.38389 2.67715 -2.05594
H -1.15702 4.742483 -0.25406
H -2.24003 3.89324 0.869812
H -2.35854 3.575692 -0.86703
H -0.20582 3.043407 1.995878
H 2.106294 2.583419 0.903195
H 3.777325 0.42776 -2.3172
H 4.62757 1.093007 -0.90277
H 4.861805 -0.60928 -1.38196

H -4.15442 1.093305 -0.083
H -5.08333 -0.85199 1.287682
H -5.11753 -1.82007 -0.19235
H -6.08853 -0.35159 -0.08798
H -3.27984 0.675047 -2.37514
H -3.80289 -1.01567 -2.28596
H -4.99693 0.284875 -2.277
H -1.37718 0.740982 -1.28198
H -3.04389 -2.38214 -0.0654
H -1.80197 -1.77846 -1.14177
H -3.16467 0.556298 1.911555
H -1.164 1.560151 1.651217
H -1.41865 -2.34095 1.843154
H -1.15215 -3.5111 0.550194
H 3.646698 -2.3847 0.538147
H 4.358895 -0.87003 1.086911
H 2.478759 -0.27211 2.415157
H 2.881046 -1.92188 2.802361
H 0.455645 -1.66699 2.711752
H 1.423322 0.492506 0.555981
H 0.797633 0.554299 -1.81462
H 2.221635 1.501607 -2.19918
H 1.460983 3.448952 -1.12001
H 0.064808 2.818736 -1.96763
H -1.40582 4.242703 1.085199
H -0.25448 4.926199 -0.0814
H -1.67035 4.000908 -0.64867
H 0.503054 2.971355 2.081835
H 2.670192 2.233519 0.830594
H 3.689148 -0.22252 -2.46173
H 4.74299 0.275831 -1.11726
H 4.607085 -1.44393 -1.5713

Table S6. Conformers and Boltzmann populations of 1β4β-**3**.

1β4β-**3a** (5.47%)				1β4β-**3b** (94.53%)			
C	-2.47035	-0.86212	-0.2012	C	2.520096	-0.64194	0.275582
C	-4.0005	-0.611	0.058619	C	4.006418	-0.18438	0.027139
C	-4.54383	0.57903	-0.74375	C	4.224002	0.520089	-1.31891
C	-4.37227	-0.47128	1.543314	C	5.014277	-1.33334	0.201119
C	-1.65679	0.317141	0.318589	C	1.69238	0.617211	0.527143
C	-2.05319	-2.17908	0.521542	C	2.008737	-1.53511	-0.89017
O	-2.37926	-1.01263	-1.61137	O	2.587609	-1.38698	1.490328
C	-1.18281	1.331771	-0.41339	C	1.062128	1.412269	-0.34504
C	-0.53224	2.599743	0.115752	C	0.486908	2.785281	-0.01617
C	-0.79508	-2.91719	0.010891	C	0.920606	-2.58828	-0.56357
C	0.545968	-2.20207	0.174437	C	-0.51934	-2.10524	-0.41896
C	1.061879	-1.5831	-1.08741	C	-0.8813	-1.64294	0.962202
O	2.310522	-1.08192	-1.22523	O	-2.01503	-0.988	1.28731
C	3.082065	-0.33134	-0.24435	C	-3.04182	-0.42939	0.41741
C	3.492497	-1.23272	0.926084	C	-3.62207	-1.52152	-0.49069
C	2.393281	-1.59517	1.921499	C	-2.77949	-1.88691	-1.70767
C	1.131964	-2.20747	1.388942	C	-1.33895	-2.22699	-1.48311
O	0.377237	-1.58701	-2.10181	O	-0.13122	-1.87727	1.900261
C	2.226353	0.894018	0.200137	C	-2.42311	0.75968	-0.37188
C	1.74877	1.770105	-0.96375	C	-1.90453	1.905454	0.502279
C	0.803698	2.929884	-0.60316	C	-1.02914	2.899146	-0.28799
C	-0.37647	2.644608	1.643763	C	1.22946	3.840963	-0.86325
O	-1.38645	3.704391	-0.27524	O	0.633417	3.106329	1.370885
O	2.923126	1.63462	1.20454	O	-3.3512	1.227531	-1.3578
C	4.335656	0.076448	-1.02507	C	-4.11755	0.024588	1.410178
H	-4.48869	-1.51544	-0.33138	H	4.213601	0.542487	0.825712
H	-5.63451	0.631768	-0.64189	H	4.093398	-0.16861	-2.16249
H	-4.29811	0.4808	-1.8032	H	5.247553	0.908389	-1.37542
H	-4.13067	1.528934	-0.38446	H	3.540739	1.363173	-1.46027
H	-5.45885	-0.36981	1.646198	H	4.916506	-2.08825	-0.58903
H	-4.06816	-1.33707	2.140868	H	4.882447	-1.82894	1.164118
H	-3.92055	0.423493	1.989053	H	6.036057	-0.93866	0.144975
H	-1.51925	0.330412	1.398851	H	1.722984	0.915979	1.574921
H	-1.93598	-1.98295	1.59502	H	1.673023	-0.91183	-1.72764
H	-2.8851	-2.88842	0.423159	H	2.864635	-2.10816	-1.26328
H	-1.44974	-1.19989	-1.85111	H	1.672726	-1.60068	1.76377

H	-1.33747	1.317114	-1.49168	H	1.012429	1.144731	-1.40102
H	-0.73869	-3.86066	0.567022	H	0.935651	-3.31425	-1.38387
H	-0.93752	-3.18367	-1.03776	H	1.218501	-3.1321	0.335759
H	3.917139	-2.14652	0.492755	H	-3.7893	-2.40531	0.137611
H	4.296355	-0.73317	1.476259	H	-4.60395	-1.19122	-0.84408
H	2.116944	-0.70539	2.508569	H	-2.82051	-1.06515	-2.438
H	2.813992	-2.29167	2.659753	H	-3.24811	-2.73993	-2.21966
H	0.561091	-2.72455	2.162976	H	-0.86889	-2.64327	-2.37575
H	1.345033	0.497366	0.709756	H	-1.58545	0.365881	-0.95288
H	2.624482	2.206055	-1.4668	H	-1.34127	1.507293	1.351209
H	1.272053	1.124198	-1.70731	H	-2.76636	2.43502	0.93265
H	1.334489	3.678339	-0.00273	H	-1.2151	2.788041	-1.36299
H	0.529166	3.425972	-1.54062	H	-1.30726	3.927177	-0.02938
H	-1.33761	2.469493	2.141727	H	0.835476	4.839352	-0.64487
H	-0.02806	3.642024	1.927731	H	1.118936	3.648716	-1.9367
H	0.345782	1.917349	2.024673	H	2.301338	3.833328	-0.63148
H	-2.25955	3.532846	0.114218	H	1.577682	3.037879	1.586459
H	3.515306	2.259729	0.757285	H	-3.97757	1.821478	-0.91401
H	4.074434	0.560634	-1.96879	H	-4.57738	-0.84477	1.890279
H	4.950176	0.758707	-0.42825	H	-3.69097	0.656982	2.192077
H	4.939024	-0.80696	-1.25601	H	-4.90651	0.580443	0.891499
